# “Let's talk about sex, inflammaging, and cognition, baby”: A meta-analysis and meta-regression of 106 case-control studies on mild cognitive impairment and Alzheimer's disease

**DOI:** 10.1016/j.bbih.2024.100819

**Published:** 2024-07-20

**Authors:** Ryan Childs, Diana Karamacoska, Chai K. Lim, Genevieve Z. Steiner-Lim

**Affiliations:** a NICM Health Research Institute, Western Sydney University, Penrith, NSW, 2751, Australia; b Faculty of Medicine, Health, and Human Sciences, Macquarie University, Macquarie Park NSW, 2190, Australia

**Keywords:** Alzheimer's disease, Mild cognitive impairment (MCI), Inflammation, Cognition, Microglia, Sex-differences, Meta-regression

## Abstract

**Background:**

Chronic inflammation is recognised as an important component of Alzheimer's disease (AD), yet its relationship with cognitive decline, sex-differences, and age is not well understood. This study investigated the relationship between inflammatory markers, cognition, sex, and age in individuals with mild cognitive impairment (MCI) and AD.

**Methods:**

A systematic review was performed to identify case-control studies which measured cognitive function and inflammatory markers in serum, plasma, and cerebrospinal fluid in individuals with MCI or AD compared with healthy control (HC) participants. Meta-analysis was performed with Hedges’ *g* calculated in a random effects model. Meta-regression was conducted using age, sex, and mini-mental status exam (MMSE) values.

**Results:**

A total of 106 studies without a high risk of bias were included in the meta-analysis including 18,145 individuals: 5625 AD participants, 3907 MCI participants, and 8613 HC participants. Combined serum and plasma meta-analysis found that IL1β, IL6, IL8, IL18, CRP, and hsCRP were significantly raised in individuals with AD compared to HC. In CSF, YKL40, and MCP-1 were raised in AD compared to HC. YKL40 was also raised in MCI compared to HC. Meta-regression analysis highlighted several novel findings: MMSE was negatively correlated with IL6 and positively correlated with IL1α in AD, while in MCI studies, MMSE was negatively correlated with IL8 and TNFα. Meta-regression also revealed sex-specific differences in levels of IL1α, IL4, IL6, IL18, hsCRP, MCP-1, and YKL-40 across AD and MCI studies, and age was found to account for heterogeneity of CRP, MCP-1, and IL4 in MCI and AD.

**Conclusion:**

Elevated levels of IL6 and YKL40 may reflect microglial inflammatory activity in both MCI and AD. Systemic inflammation may interact with the central nervous system, as poor cognitive function in individuals with AD and MCI was associated with higher levels of serum and plasma proinflammatory cytokines IL6 and TNFα. Moreover, variations of systemic inflammation between males and females may be modulated by sex-specific hormonal changes, such as declining oestrogen levels in females throughout the menopause transition. Longitudinal studies sampling a range of biospecimen types are needed to elucidate the nuances of the relationship between inflammation and cognition in individuals with MCI and AD, and understand how systemic and central inflammation differentially impact cognitive function.

## Highlights

• Raised central and peripheral pro-inflammatory cytokines found in AD and MCI.
• Pro-inflammatory cytokines correlated with cognitive function.
• Sex differences found in cytokines associated with microglial activity.
• Ageing associated with changes in pro- and anti-inflammatory activity.

## 1 Introduction

Alzheimer's disease (AD) is the most common cause of dementia and is characterised pathologically by the accumulation of extracellular amyloid-β (Aβ) plaques and intracellular neurofibrillary tangles of hyperphosphorylated tau (Knopman et al., 2021). AD can be preceded by mild cognitive impairment (MCI), an early symptomatic prodromal phase of cognitive decline that increases dementia risk (Petersen, 2016). The current gold standard diagnostic modalities for AD include expensive and largely inaccessible imaging modalities such as positron emission tomography (PET) and invasive collections of cerebrospinal fluid (CSF) by lumbar puncture to measure levels of Aβ and hyperphosphorylated tau (Dubois et al., 2021). Research into a wider range of pathological mechanisms of AD and MCI (e.g., inflammation) may allow the development of more accessible, inexpensive, and less invasive diagnostic modalities, such as collections of peripheral blood samples, which could be used for early diagnosis and intervention.

Systemic inflammatory markers are a promising example of less invasive diagnostics and multiple studies have uncovered differences in the peripheral inflammatory profile of individuals with MCI and AD compared to cognitively healthy individuals (Bradburn et al., 2019; Shen et al., 2019; Su et al., 2019). However, these studies have shown a high level of unexplained heterogeneity in their results. This heterogeneity is evident not only in studies of MCI and AD, but also in meta-analyses investigating peripheral inflammatory markers in cohorts of older adults with healthy cognition, frailty, Parkinson's disease, and multiple sclerosis (Bai et al., 2019; Leonardo and Fregni, 2023; Qu et al., 2023; Xu et al., 2022). These findings highlight the intricate nature of the inflammatory response in different disease states and stress the importance of identifying sources of heterogeneity to better understand them. Cognitive decline, which reflects clinical disease progression in AD, may be an important source of heterogeneity in these measures of circulating inflammatory markers (Holmes, 2009; Raket, 2020). Further, age is recognised to be the most important non-modifiable risk factor in the development of AD (Knopman et al., 2021). As humans age, inflammatory mechanisms become dysregulated through a process described as “inflammaging” which may confer a greater risk of neurodegenerative diseases including AD, Parkinson's disease, and amyotrophic lateral sclerosis (Cianciulli et al., 2022; Franceschi et al., 2007).

Biological sex may offer another important confounding factor influencing the heterogeneity of these studies. Reduced levels of oestrogen through the perimenopausal transition period is associated with increased levels of systemic inflammation, which promotes central neuroinflammation and may contribute to the increased risk of AD in female populations (McCarthy and Raval, 2020; Nichols et al., 2022). Sex-specific differences have been noted on a cellular level within the CNS as microglia and astrocyte response to pathology in neurodegenerative disease is influenced by gonadal hormones and neuroactive steroid metabolites (Chowen and Garcia-Segura, 2021).

Centrally, chronic neuroinflammation occurs in AD following the prolonged activation of glial cells by plaques and tangles (Dani et al., 2018). Upregulation of pro-inflammatory cytokines and oxidative species perpetuates neurotoxicity and neurodegeneration (Kempuraj et al., 2016). Circulating cytokines, referring to those found in the serum and plasma, may also interact with the central nervous system (CNS) and higher levels of peripheral pro-inflammatory cytokines have previously been shown to be associated with cognitive decline (Kipinoinen et al., 2022). Mechanisms by which this may occur include cytokine induced disruption of, and communication across, the blood brain barrier (Lyman et al., 2014). Once in the CNS, these inflammatory molecules may promote microglial and astrocyte activation and subsequently drive neuroinflammation (Reale et al., 2009). The gut-brain-axis is another important source of peripheral inflammation, as gut dysbiosis is associated with an increased peripheral inflammatory profile and increased risk of AD (Bettcher et al., 2021). Routes of communication between the peripheral and central immune systems also include the vagus nerve and transport across circumventricular organs (Bettcher et al., 2021).

Previous meta-analyses have shown that serum levels of interleukin-1β, (IL1β), IL6, and tumour necrosis factor α (TNFα) are higher in AD compared to healthy controls (HC) and MCP-1 is higher in MCI compared to HC (Lai et al., 2017; Su et al., 2019). Nonetheless, it remains uncertain whether inflammatory activity in the peripheral and central immune systems directly correlate with cognitive function in these conditions. The results of these previous meta-analyses have shown a high level of heterogeneity, which we hypothesise might arise from participant characteristics, notably the variation of the cognitive status, age, and the biological sex of enrolled participants. Therefore, in this study we performed a meta-analysis with robust meta-regressions to investigate how these three key factors: cognition, age, and sex may be associated with differences in the levels of peripheral and central inflammatory markers in AD and MCI. To do so, this study only focused on reports which involved simultaneous measurements of inflammation and cognition.

## 2 Methods

This systematic review was registered through PROSPERO (CRD42023454033). This review followed the methods described in the Preferred Reporting Items for Systematic Reviews and Meta-Analyses (PRISMA) statement (Moher et al., 2009).

### 2.1 Eligibility criteria

A scoping review was performed to review the literature and define the PICOS including the population, prognostic factors, comparison, outcomes, and study design, as well as the eligibility criteria, that will be used for this systematic review.

• Population: individuals living with MCI or AD
• Prognostic factor: markers of inflammation from blood, CSF, or urine
• Comparison: appropriate control group (e.g., age-matched cognitively normal controls)
• Outcomes: cognitive function
• Study design: observational cross-sectional studies

Inclusion criteria included peer-reviewed original research articles that: a) report quantitatively measured inflammatory factors (e.g., ELISA) measured *in vivo* AND measures of cognition taken at the same timepoint; and b) include people with MCI or dementia and an appropriate control group. Studies were excluded if they did not meet inclusion criteria, were not journal articles, were not written in English, or if their full text was not available. No date restrictions were applied. Articles were screened by titles and abstracts in Endnote to find those that meet eligibility criteria.

### 2.2 Systematic search strategy

PubMed, PsycINFO, Scopus, and Web of Science were searched for relevant articles on June 31, 2022 and an updated search was completed on the 4 September of 2023 to allow for article finalisation. Search strategies used in these databases can be found in Supplementary Material 1 Tables S1–3. Reference lists of previous systematic reviews were also screened for articles which may have been eligible for inclusion.

### 2.3 Data extraction

Data extracted from eligible articles included first author, publication date, location, sample sizes, participant demographics (age, diagnosis type and criteria, sex, years of education, body mass index), measures and findings related to circulating and CSF inflammatory markers and cognition, 95% confidence intervals, and confounders used for adjustment in the analysis.

### 2.4 Risk of bias

Risk of bias was assessed by a checklist composed of items retrieved from QUADOMICS, an adaptation of the Quality Assessment of Diagnostic Accuracy Assessment (QUDAS) tool (Lumbreras et al., 2008), and the Joanna Briggs Institute checklist for analytical cross sectional studies (Joanna Briggs Institute, 2017). Items on this 11-point checklist were scored as yes (Y) = 1, no (N) = 0, unknown (U), or not applicable (N/A). For N/A items, the total score was adjusted to reflect applicable items. Studies which lost 3-points or more were deemed at high risk of bias. Additionally, Begg's funnel plot and Egger's test were inspected for assessment of bias (Begg and Mazumdar, 1994; Egger et al., 1997). Papers at high risk of bias were excluded from meta-analysis and meta-regression.

### 2.5 Data analysis and synthesis

Meta-analyses were performed using random effects models to calculate Hedges’ *g* effect sizes using means and standard deviations of inflammatory markers as retrieved from at least 3 individual articles or calculated primarily from medians and inter-quartile ranges. Random effects model was selected due to the heterogeneity of methods identified in individual articles. Heterogeneity was assessed using *Q*-tests and *I*^2^ index to measure the variance between studies. Studies with an *I*^2^ of greater than 75% were considered to have high heterogeneity, while an *I*^2^ of between 25 and 75% were considered to have moderate heterogeneity, and those with an *I*^2^ of less than 25% were considered to have low heterogeneity. Inverse-variance weighted meta-regression analyses were performed to explore potential heterogeneity with regression of standard mean averages against inflammatory markers, sex, age, years of education, and measures of cognition.

Meta-regressions were performed using three models with age, sex and MMSE values against the Hedges' *g* effect size. For the models of age and MMSE, the means for each group were used in the regression. For the model of sex, the portion of female participants as a percentage was used in the regression. These models were constructed by grouping AD and MCI separately from healthy control (HC) participants to identify the unique effect of the moderators on each group. Regressions were also performed to assess if sample type influenced Hedges’ *g*.

## 3 Results

### 3.1 Search

**Fig. 1:**
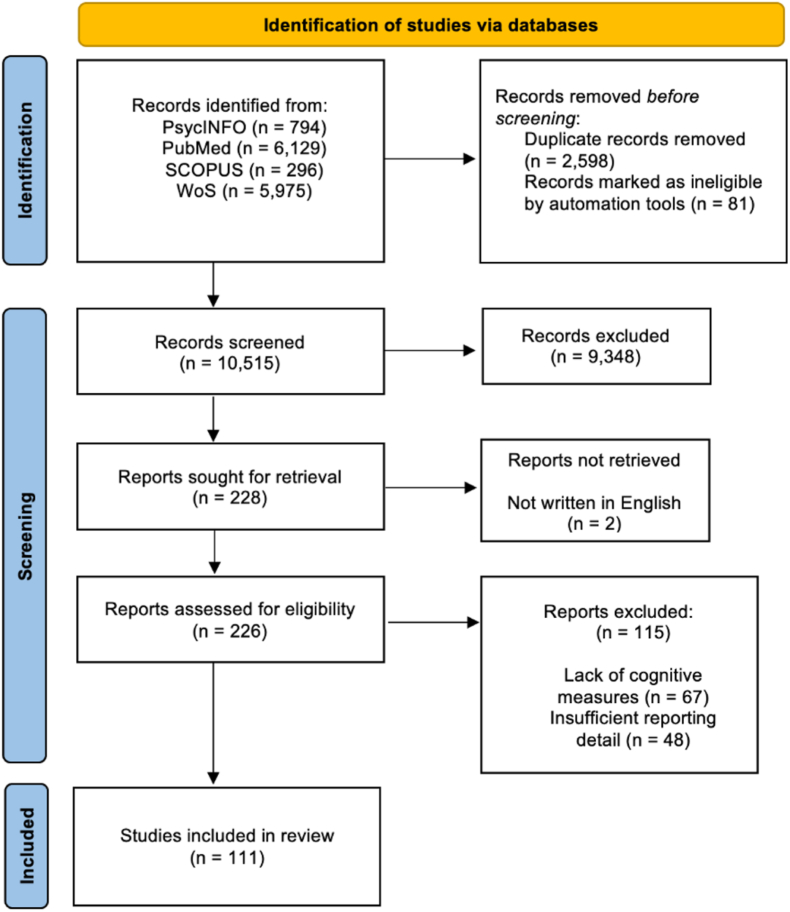
PRISMA flowchart.

The search strategy yielded a total of 11,986 records (Fig. 1). Of these, 2481 were removed as either duplicates or being automatically identified as an ineligible record type (book, book chapter, dissertation, etc.). Screening the remaining records by title and abstract identified 156 records for retrieval, though 2 of these were not retrieved as they were not published in English. After screening the full reports, 111 studies were included in the review, which included 38 inflammatory markers measured from serum, plasma, or CSF. Markers measured in 3 or more eligible studies were included in meta-analysis, giving a total of 18 markers in AD and HC studies and 16 in MCI and HC studies.

### 3.2 Study characteristics

Of the total 111 studies that were identified, 37 included measures of inflammatory markers from plasma, 51 from serum, 27 from CSF, and 1 from urine (Supplementary Material 1 Table s4). Studies which measured inflammatory markers in serum and plasma were analysed together, while studies which measured markers in CSF were analysed separately. Of the studies which measured serum and plasma, 82 included comparisons between AD and HC groups while 43 included comparisons of MCI and HC groups. Together, these studies included a total of 15,925 participants including 4832 AD, 3291 MCI, and 7802 HC participants. Of the studies which measured CSF, 24 included comparisons of AD and HC groups, while 15 included comparisons of MCI and HC groups. These CSF studies included 1047 AD, 705 MCI, and 974 HC participants.

All studies included cross-sectional data of inflammatory markers and measures of cognitive function taken at the same time point. Cognitive function was measured most commonly using the MMSE or the MoCA, with other studies using a battery of neuropsychological testing.

### 3.3 Risk of bias

The risk of bias assessment identified six studies with high risk of bias, and these were removed from the subsequent analyses (Supplementary Material 2). The remaining 106 articles were not found to be at a high risk of bias and were included in the study.

### 3.4 Hedges’ *g* meta analysis in AD and MCI

Inflammatory markers in serum, plasma, and CSF were compared for studies assessing those with AD or MCI compared to a HC group (Supplementary Material 1 Tables s5); plasma and serum were assessed in combination, whereas CSF was assessed separately. Forest plots for each meta-analysis are presented in Supplementary Material 3 Figs. S1–S38. Combined serum and plasma analysis found that IL1β (*p* = 0.001), IL6 (*p* = 0.010), IL8 (*p* = 0.046), IL18 (*p* = 0.007), CRP (*p* = 0.029), and hsCRP (*p* < 0.001) were significantly raised in serum and plasma in people with AD compared to HC (Table 1).

**Table 1:** Statistically significant findings from meta-analyses.

Sample	Marker	Studies	*N*	Hedges' *g* (95% CI)	*z*	*p-*value	*Q* (*df*)	*τ*^2^	*I*^2^	Egger's regression 2-tailed *p-*value
AD										
Serum and plasma	IL1β	25	AD = 1012 HC = 937	0.680 (0.262–1.097)	3.192	0.001	418.207	1.045	94.261	0.017
	IL6	25	AD = 1103 HC = 1095	0.381 (0.092–0.670)	2.583	0.010	244.848 (24)	0.469	90.198	0.0572
	IL8	10	AD = 384 HC = 407	0.798 (0.013–1.583)	1.992	0.046	199.247 (9)	1.491	95.483	0.617
	IL18	10	AD = 455 HC = 493	1.180 (0.322–2.039)	2.693	0.007	298.710 (9)	1.851	96.987	0.019
	CRP	13	AD = 1107 HC = 1705	0.355 (0.037–0.674)	2.186	0.029	147.715 (12)	0.277	91.876	0.074
	hsCRP	8	AD = 1095 HC = 745	0.649 (0.284–1.013)	3.483	<0.001	72.877 (7)	0.228	90.395	0.234
CSF	MCP-1	5	AD = 199 HC = 220	0.793 (0.168–1.417)	2.489	0.013	30.263 (4)	0.427	86.782	0.0378
	YKL40	7	AD = 371 HC = 388	1.155 (0.872–1.438)	8.010	<0.001	15.723 (6)	0.085	61.840	0.991
MCI										
CSF	YKL40	7	MCI = 371 HC = 388	0.950 (0.584–1.316)	5.085	<0.001	30.913 (6)	0.191	80.591	0.066

In CSF, YKL40 (*p* < 0.001) and MCP-1 (0.013) were raised in AD compared to HC (Table 1). YKL40 was also raised in MCI compared to HC (*p* < 0.001) (Table 1). There were no other inflammatory markers for any of the specimen types that significantly differed between AD and HC or MCI and HC.

### 3.5 Heterogeneity

Egger's regression test found potentially significant publication bias (*p* < 0.05) for serum measures of IL1β and IL18 and CSF MCP-1 in studies including AD and HC participants (Table 1), as well as serum and plasma measures of IL1α in studies of MCI and HC participants (Supplementary Material 1 Table s5). No other markers or specimen types for AD or MCI were identified to have potential publication bias by the Egger's regression test. As shown in Table 1, CSF measures of YKL40 in both AD had moderate heterogeneity (*I*^2^ 25–75%), while all other markers showed high heterogeneity (*I*^2^ > 75%). Details of heterogeneity for all markers included in meta-analyses can be found in Supplementary Material 1 Table s5.

### 3.6 Meta-regression

#### 3.6.1 Pro-inflammatory cytokines correlated with cognitive function in MCI and AD

Meta-regression analyses found that MMSE values in studies of AD and HC participants correlated with differences in the pro-inflammatory markers IL1α and IL6 (Table 2). Studies which measured higher serum and plasma IL6 levels were found to have lower MMSE scores (*p* < 0.001). On a group level, this finding was significant in HC (*p* = 0.002) but not in AD (*p* = 0.169) (Supplementary Material 3 Fig. S39A). Similarly, studies with higher CSF measures of IL6 were also found to have lower MMSE values (*p* < 0.001) (Table 2). Again, this was significant at a group level in HC (*p* < 0.001) and not in AD (*p* = 0.441) (Supplementary Material 3 Fig. S39B). In contrast to these results, studies which measured higher levels of IL1α were associated with increased cognitive function as measured by MMSE (*p* < 0.001) (Table 2). These results were also significant in HC groups (*p* < 0.001) and not in AD (*p* = 0.206) (Supplementary Material 3 Fig. S39C).

**Table 2:** Regression of inflammatory marker Hedges’ *g* measured in serum, plasma, and CSF against the models of MMSE, Age, and Sex. *Indicates model uses CSF measures.

			Test of the model			Accounted variance	Goodness of fit		
AD									
Marker	Model	Studies	*Q*	df	*p*	*r*^2^	*Q*	df	*p*
IL1α	MMSE	6	37.19	2	<0.001	1	2.26	3	0.521
	Sex	6	7.60	2	0.022	0.65	8.58	3	0.036
IL6	MMSE	24	14.08	2	<0.001	0.24	170.17	21	<0.001
	MMSE*	6	93.14	2	<0.001	0.96	7.07	3	0.070
IL18	Sex	10	21.75	2	<0.001	0.49	122.99	7	<0.001
CRP	Age	12	6.19	2	0.045	0.12	75.18	9	<0.001
MCP-1	Age*	5	27.42	2	<0.001	1	2.03	2	0.363
	Sex*	5	24.98	2	<0.001	0.99	2.09	2	0.351
MCI									
IL4	Age	6	11.99	2	0.003	0	50.14	3	<0.001
	Sex	5	26.04	2	<0.001	0.58	16.56	2	0.0003
IL6	Sex	15	6.56	2	0.038	0.33	31.28	12	0.0018
IL8	MMSE	5	22.98	2	<0.001	1	1.15	2	0.5617
hsCRP	Sex	5	12.29	2	0.002	1	1.76	2	0.4146
TNFα	MMSE	13	6.74	2	0.034	0.19	148.4	10	<0.001
YKL40	Sex*	7	10.03	2	0.007	0.79	7.1	4	0.1308

In studies including MCI and HC participants, the pro-inflammatory cytokines IL8 and TNFα were found to correlate with cognitive function (Table 2). Studies which measured higher circulating TNFα levels were associated with reduced MSME values (*p* = 0.034) however, this was not apparent at the MCI or HC group level (Supplementary Material 3 Fig. S39D). Raised serum and plasma IL8 levels were also associated with reduced MMSE scores (*p* < 0.001) (Table 2), though this was only significant in the HC group (*p* < 0.001) and not the MCI group (*p* = 0.268) (Supplementary Material 3 Fig. S39E).

#### 3.6.2 Sex-specific differences found in cytokines associated with microglial activity in MCI and AD

Regression analyses revealed sex-specific differences in the levels of IL1α, IL4, IL6, IL18, hsCRP, MCP-1, and YKL-40, all of which are implicated in microglial activity. In AD and HC studies that included more female participants, there were reduced levels of the circulating IL1 family cytokines IL1α (*p* = 0.022) and IL18 (*p* < 0.001), while CSF levels of MCP-1 were raised (*p* < 0.001) (Table 2). For IL1α, this was significant in both AD (*p* = 0.011) and HC (*p* = 0.006) groups (Supplementary Material 3 Fig. S40A). For IL18 the model of sex was only significant in the AD group (*p* = 0.035) and not in HC groups (*p* = 0.995) (Supplementary Material 3 Fig. S40B). For MCP-1 this was significant within the HC groups (*p* < 0.001) but not AD groups (*p* = 0.807) (Supplementary Material 3 Fig. S40C).

In MCI and HC studies, sex-specific differences were found in IL4, IL6, hsCRP, and YKL-40. Studies which included higher proportions of female participants with MCI had reduced levels of circulating IL6 (*p* = 0.038) and hsCRP (*p* = 0.002), while circulating IL-4 (*p* < 0.001) and CSF YKL-40 (*p* = 0.007) were raised (Table 2). For IL6, this was not significant at a group level (Supplementary Material 3 Fig. S40D), while for hsCRP, this was significant in both MCI (*p* = 0.036) and HC (*p* = 0.001) groups (Supplementary Material 3 Fig. S40E). For IL4 on a group level there was significance in both the MCI (*p* < 0.001) and HC groups (*p* < 0.001) (Supplementary Material 3 Fig. S40F) and in YKL-40 there was significance in MCI groups (*p* = 0.002) and not in HC (*p* = 0.054) (Supplementary Material 3 Fig. S40G).

#### 3.6.3 Age associated with reduced pro-inflammatory markers in AD and increased anti-inflammatory markers in MCI

In AD studies that included participants of more advanced age, it was found that the circulating levels of CRP (*p* = 0.045) and CSF MCP-1 (*p* < 0.001) were reduced (Table 2). For CRP on a group level this was only significant in the HC groups (*p* = 0.035) and not the AD groups (*p* = 0.247) (Supplementary Material 3 Fig. S41A). For MCP-1, this model was significant in both AD (*p* < 0.001) and HC groups (*p* < 0.001) (Supplementary Material 3 Fig. S41B).

In MCI studies, it was found that increased age was associated with increased serum and plasma levels of the anti-inflammatory cytokine IL4 (*p* = 0.003) (Table 2). This was significant in the MCI groups (*p* = 0.002), but not in the HC groups (*p* = 0.090) (Supplementary Material 3 Fig. S41C).

## 4 Discussion

The aim of this study was to conduct a meta-analysis and meta-regression that assessed the relationship between inflammation and age, sex, and cognition in studies on AD and MCI compared to a control group. In total, this review included serum, plasma, and CSF measures of inflammatory markers from 18,145 individuals (5625 with AD, 3907 with MCI, and 8613 HC) across 106 studies. Several measures of cognition were extracted during the review process, however, only the MMSE was identified consistently enough between the included studies to perform viable regressions. Of studies which included AD and HC participants, 94.9% reported the participant characteristics of age and sex. Of studies which included MCI and HC participants, 96.5% reported participant age and 91.2% reported participant sex.

Differences in circulating serum and plasma inflammatory markers, including IL1β, IL6, and CRP were identified in AD compared to HC participants. Measures of YKL-40 and MCP-1 in CSF were also raised in AD compared to HC. Only CSF measures of YKL-40 were found to be raised in MCI and HC groups. These results are in line with previous meta-analyses which have provided a comprehensive review of the difference in inflammatory markers in AD, MCI, and HC (Shen et al., 2019; Su et al., 2019). Heterogeneity among all significant inflammatory markers were high, aside from YKL-40 in AD, which was only moderate. Meta-regressions were performed to identify potential reasons for this. In both AD and MCI, it was identified that the Hedges’ *g* effect size calculated from multiple inflammatory markers could be correlated with age, sex, and MMSE.

To the best of our knowledge, this is the first study on inflammatory markers in AD and MCI to have reported the data of meta-regressions on the variables of age, sex, and cognitive function individually for MCI, AD, and control groups. In this study, regression of MMSE on Hedges’ *g* accounted for a significant proportion of the variance in IL1α and IL6 in AD, and IL8 and TNFα in MCI. Age was found to influence levels of CRP and MCP-1 in AD studies, and IL4 in MCI studies. The regression also found a linear relationship between the percentage of women enrolled in these studies and the difference between IL1α, IL18, and MCP-1 in AD, and IL4, IL6, hsCRP, and YKL-40 in MCI.

### 4.1 Pro-inflammatory cytokines correlated with cognitive function in MCI and AD studies

Cognition was found to account for some of the variance in the Hedges' *g* meta-analyses of IL6 and TNFα. Lower MMSE values were found to be significantly associated with a higher Hedges’ *g* effect sizes for IL6 in AD and HC studies and for TNFα in MCI and HC studies. For IL6, this correlation was observed in both serum and CSF measures. Further, it was found that studies with higher MMSE values in HC participants had smaller effect sizes for IL1α in AD compared to HC and for IL8 in MCI compared to HC. Lai et al. (2017) and Shen et al. (2019) found similar evidence that MMSE values explained some variance of the standard mean difference of circulating IL6 levels compared between AD and HC participants, but Shen et al. did not make this observation in studies including MCI groups (Shen et al., 2019). Circulating IL6 is implicated in cognitive decline even in cohorts without clinical dementia, in line with our HC sub-group finding (Bradburn et al., 2018). Similarly, raised circulating levels of TNFα have been associated with decreased functional connectivity within the hippocampus of individuals with amnestic MCI and AD (Magalhães et al., 2018). Longitudinal studies have also shown that increased peripheral and CSF IL6 and TNFα have been associated with increased risk of progression from cognitively unimpaired to MCI and to AD (Abe et al., 2020; Contreras et al., 2022; Gross et al., 2019; Oberlin et al., 2021). Furthermore, disease states with systemic inflammation including cancer, type 2 diabetes mellitus, and systemic lupus erythematosus have found a close relationship with inflammatory activity and cognitive dysfunction (Ho et al., 2018; Országhová et al., 2021; Yang et al., 2020).

Neuroinflammation is recognised to disrupt cognitive function in a variety of neuropsychiatric disease states through alterations of microglial activity (Fourrier et al., 2019). In neurodegenerative diseases, including Parkinson's disease, amyotrophic lateral sclerosis, and Huntington's disease, neuroinflammation is not just an outcome, but a key pathophysiological mechanism driving disease progression (Zhang et al., 2023a, Zhang et al., 2023b). While transient microglial activation is necessary for the protective function of the central immune system, such as in the process of microglial Aβ clearance, but anti-inflammatory cytokines produced in the CNS such as IL4, IL10, and TGFβ are required to limit excessive neuroinflammatory activity (Leng and Edison, 2021). As microglia become chronically activated, they release cytokines including TNFα, IFNγ, and IL1β in a viscous cycle of self-propagating neuroinflammation which incites irreversible neuronal damage while increasing Aβ deposition and tau phosphorylation (Liu et al., 2023). In fact, increased inflammatory activity in both the central and peripheral systems has been correlated with increased amyloid and tau pathology and cognitive changes in AD (Cullen et al., 2021). These processes impair BBB integrity and lymphatic drainage which leads to CNS invasion of adaptive immune cells and clonal expansion of CD8^+^ T cells. These factors may also play a role in neurodegeneration and cognitive impairment (Jorfi et al., 2023). Although microglia are often considered the principal orchestrators of neuroinflammation, neurons, astrocytes, and endothelial cells can also produce pro-inflammatory cytokines, including IL6 and YKL-40, in response to insults to the CNS (Erta et al., 2012; Querol-Vilaseca et al., 2017). Recognising this broader involvement is crucial to understanding neuroinflammation as a coordinated event between multiple cell types.

Aside from inducing frank neuron cell death, microglia indirectly modulate synaptic plasticity through the production of cytokines, and overproduction of IL1β, IL6, and TNFα may disrupt important mechanisms regulating memory and cognition such as long-term potentiation (Fourrier et al., 2019). Furthermore, microglia and astrocytes modulate synaptic activity through mechanisms including synaptogenesis, spine formation, and synaptic pruning (Lecca et al., 2022). By disruption of these mechanisms, glial dysfunction may play an important role in cognitive changes in states of chronic neuroinflammation (Lecca et al., 2022). In line with this, chronic microglial activation with release of IL6 and TNFα is associated with reduced hippocampal neurogenesis, which manifests in a rat model of AD with reduced spatial learning and memory (Bassani et al., 2017). Raised peripheral levels of IL6 in AD have also been associated with inflammatory activity within the hippocampus, reduced hippocampal volume and reduced MMSE performance (Lyra E Silva et al., 2021). Together, this evidence provides support for our findings that levels of IL6 in 10.13039/100020014AD and TNFα in MCI are associated with reduced cognitive function.

**Fig. 2:**
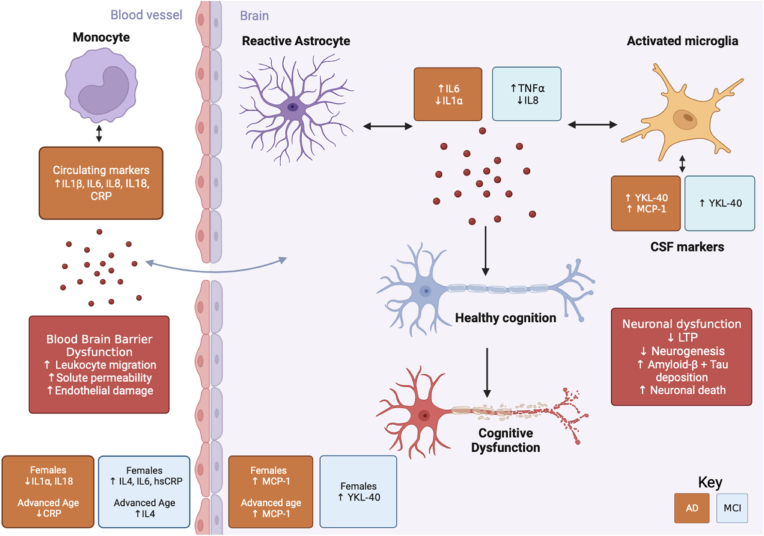
Inflammatory markers in Alzheimer's disease (AD), mild cognitive impairment (MCI), and healthy controls (HC) and their relationship with cognition, sex, and age. In the peripheral circulating system and cerebrospinal fluids (CSF) raised levels of inflammatory markers may cause blood brain barrier dysfunction mediating central-peripheral immune crosstalk. In the central nervous system (CNS) these inflammatory mediators can induce reactive astrogliosis and microglial activation to incite and drive neuroinflammation which may induce neuronal dysfunction. The results of this study suggest that raised interleukin 6 (IL6) and raised tumour necrosis factor α (TNFα) are associated with cognitive dysfunction in AD and MCI respectively as measured by the mini mental status examination (MMSE). Sex-specific differences and age showed relationships with inflammatory markers in both AD and MCI within the peripheral circulating and CSF. Left: peripheral circulating system; Right: central nervous system. Orange boxes indicate results retrieved from AD and HC studies; Blue boxes indicate MCI and HC studies; Red boxes indicate hypothesised mechanisms underlying relationships. CRP, C-reactive protein; hsCRP, high sensitivity CRP; LTP, long-term potentiation; MCP-1, monocyte chemoattractant protein-1; YKL-40, Chitinase-3-like protein 1.

Indeed, ascertaining the directionality of the communication of these cytokines across the BBB is difficult. IL6 and TNFα may rise in systemic inflammation wherein they interrupt the BBB and enter the CNS to induce microglial activation (Xie et al., 2022). On the other hand, previous evidence has shown that IL6 and TNFα efflux from the CNS to the blood, raising circulating levels of these cytokines (Banks, 2015). Fig. 2 offers a visual depiction to summarise these results and the potential mechanisms by which raised pro-inflammatory markers in the circulating system and the CNS might impair cognitive function.

Even prior to the onset of MCI and AD, neuroinflammation has been shown to influence cognition in healthy older adults (Sartori et al., 2012). Here it was shown that higher MMSE values in HC groups were associated with a lower Hedges’ *g* value in IL1α compared to AD groups and IL8 compared to MCI groups. IL8 is a chemokine which recruits macrophages and neutrophils to the CNS and raised levels in mice has been associated with impaired hippocampal long-term potentiation and reduced memory performance in older adults (Baune et al., 2008; Capogna et al., 2023; Xiong et al., 2003). IL1α is closely associated with the early innate immune response, and raised serum levels in HC participants over years have been associated with increased risk of incident dementia (Brough and Denes, 2015; Kim et al., 2018). Taken together, these results support that central-peripheral immune crosstalk may influence cognition across the cognitive continuum from older adults to MCI and 10.13039/100020014AD (Sun et al., 2022).

### 4.2 Sex-specific differences in microglia-mediated cytokines in MCI and AD

Regression analyses found a correlation between the proportion of AD and MCI female participants and levels of the inflammatory markers IL1α, IL4, IL6, IL18, hsCRP, MCP-1, and YKL-40, which are each associated with microglial activity. Supplementary Material 1 Table S6 provides details of how these cytokines interact with the immune system both centrally and peripherally. Studies which included more women with AD were found to have reduced levels of the IL1 family cytokines IL1α and IL18. This may be a novel finding, as previous studies have reported that IL1α and IL18 are either raised in females with AD compared to males, or that there is no difference (Chen et al., 2014). Previous studies have shown that oestradiol and progesterone have a dose-dependent effect on reducing IL1 cytokine expression from peripheral monocytes, suggesting sex-specific mechanisms may underly differences in IL-1 production (Morishita et al., 1999). Sex-differences in IL1α have been reported in a rodent models of cerebral amyloid angiopathy, where female mice have shown poorer cognitive performance than their male counterparts as well as a reduced peripheral levels of IL1α (Maniskas et al., 2021). Those authors suggest that as IL1α is associated with microglial activation, reduced levels may confer an increase in cerebral amyloid-β and so inducing a more severe cognitive dysfunction (Maniskas et al., 2021). Italiani et al. (2018) proposed that IL18 levels rise during the prodromal MCI phase, inducing neuroinflammation, and proceed to fall in AD as the inhibitor molecule IL18 binding protein is upregulated. Our results suggest that this pathogenic mechanism might be emphasised in females with AD. Further, the regression model of sex on MCP-1 found that studies which included more female participants had lower levels of CSF MCP-1 in the HC groups. MCP-1 is associated with the microglial response to Aβ (Supplementary Material 1 Table S6). In inflammatory states MCP-1 recruits microglia and permits monocytes migration to the CNS (D'Mello et al., 2009). These features may reflect an important sex-specific difference in healthy female brains with reduced microglial activity (Sokolova et al., 2009).

In MCI, studies which included more women were associated with higher levels of IL4 and YKL-40, and lower levels of hsCRP. Sex also accounted for some variance in the effect sizes of IL6 in MCI and HC studies, however, there was no significant difference at the group level. YKL-40 has been proposed as a pro-inflammatory molecule associated with microglial activation in amyloid plaques (Y. Zhang et al., 2023a, Zhang et al., 2023b). Sex and genetic factors have been identified as contributors which may influence the way YKL-40 is expressed during MCI and AD (Baldacci et al., 2019). Increased levels of YKL-40 in females with MCI may reflect an increased neuroinflammatory activity during a prodromal phase of AD. A similar relationship was found in circulating levels of IL4 in MCI, where studies including more women with MCI had higher IL4. As an anti-inflammatory cytokine, IL4 is thought to be protective in AD by augmenting microglial phagocytic capacity for Aβ (Tang et al., 2019). It has been previously reported that women with MCI have a higher burden of Aβ compared to age-matched males (Wang et al., 2023). These findings may provide a mechanistic basis for the raised levels of YKL-40 and IL4 in women with MCI compared to men. Interestingly, an opposing relationship was found in the levels of hsCRP with studies including more female participants with MCI having lower levels of circulating hsCRP. This finding is in line with previous evidence of raised CRP in males with MCI compared to females (Trollor et al., 2010). CRP is found within Aβ plaques and is understood to activate the complement C system (Strang et al., 2012). Aβ plaques dissociate pentameric CRP into CRP monomers which are able to fixate C1q and drive cortical inflammation (Strang et al., 2012). In line with this, reduced levels of CRP have been associated with impaired capacity for microglia to clear Aβ in AD (Hegazy et al., 2022). Therefore, the evidence here may suggest that females with MCI with lower hsCRP levels have less capacity for Aβ clearance.

While these results provide some evidence that sex influences the circulating inflammatory markers in AD and MCI, it is imperative for future studies to include analyses stratified by sex to entirely elucidate these mechanisms. Elucidating these mechanisms will allow development of precision medicine with individualised approaches to diagnosis, monitoring, and management of MCI and AD (Ferretti et al., 2018).

### 4.3 Age associated with reduced pro-inflammatory markers in AD and increased anti-inflammatory markers in MCI

Age was found to account for variance of the differences in circulating levels of CRP and CSF levels of MCP-1 in AD studies. In MCI studies, older MCI groups were associated with higher levels of circulating IL4 compared to HC. Previous meta-regressions have not found associations of inflammatory markers with age, and one study identified that age may account for some of the variance found among CSF measures of YKL-40 in MCI studies (Lai et al., 2017; Shen et al., 2019). Different findings may be accounted for here in that this study analysed a subset of the literature, only including studies with measures of cognition that had low risk of bias.

In studies of CRP, when HC individuals were older, the effect size became smaller. Studies with older AD participants also had a smaller effect size, however this was not statistically significant in our analysis. This finding is in line with previous evidence, as CRP levels have been shown to rise in healthy older adults while CRP levels tend to reduce in AD (De Almeida Roediger et al., 2019; Nilsson et al., 2011). A contrasting effect of CRP levels can therefore be found across the lifespan. In midlife, a higher circulating level of CRP is associated with an increased risk of AD, while in more advanced age groups, reduced CRP levels are associated with a higher risk of AD (Hegazy et al., 2022; O'Bryant et al., 2010). Hegazy et al. (2022) suggested that as CRP levels decline, microglia become less efficient at clearing Aβ and so an increased risk of AD occurs with lower levels of CRP.

In studies which measured MCP-1, increasing age of AD participants was associated with a reduced effect size, while the effect size increased with older HC participants. In line with these findings, longitudinal increases in plasma MCP-1 were associated with reduced cognitive function in cognitively normal individuals (Bettcher et al., 2019). Similarly, raised plasma MCP-1 in MCI and AD has been found to have prognostic value by providing insight into the rate of cognitive decline in these pathologies (Lee et al., 2018).

IL4 was found to have a positive correlation with age in MCI individuals. This may be a compensatory mechanism to reduce neuroinflammatory activity, as previous evidence has shown that raised IL4 levels in MCI have been associated with a protective effect against hippocampal age related atrophy (Boccardi et al., 2019). In sum, ageing is one of the key non-modifiable risk factors for 10.13039/100020014AD, and our results implicate age-related disruption of the immune system as one of the underlying mechanisms contributing to this risk factor, supporting the inflammaging hypothesis (Franceschi et al., 2007; Knopman et al., 2021).

### 4.4 Strengths and limitations

Strengths of this study included the novel approach to meta-regression analysis. By including group-level meta-regression outputs, a detailed understanding of the relationship between inflammatory markers and cognition in AD and MCI can be obtained. Further, the inclusion of a wide variety of inflammatory markers, such as cytokines and chemokines involved in the innate and adaptive immune systems, measured from both peripheral and central systems allowed for a thorough review of the relationship between inflammatory activity and cognition in AD and MCI. The large sample size provides reliability in the results presented.

This meta-analysis focused on a subset of the literature and used meta-regression to investigate cognition and systemic inflammation, and so studies which did not include a quantifiable measure of cognition or had high risk of bias were not included. As a result, the meta-analysis does not encompass the entire literature on inflammation and AD or MCI at this time-point, as has been done in previous studies (Lai et al., 2017; Su et al., 2019). This should be taken into consideration when interpreting data from the Hedges’ *g* meta-analysis, as well as meta-regressions under the models of age and sex. 10.13039/100014337Furthermore, this review only included articles with cross-sectional data, which does not provide robust evidence to support more conclusive relationships over time between age, cognition and systemic inflammation in 10.13039/100020014AD and MCI. Alternatively, longitudinal data could provide a more comprehensive understanding of the change in systemic inflammation over time. Longitudinal data would be particularly advantageous as inflammatory markers are subject to change during both acute bouts of physiological stress and across chronic disease states which together can make cross-sectional data difficult to interpret. The low number of studies included for some inflammatory markers made it not possible to perform meta-regression to explore the causes of heterogeneity in the meta-analysis. Finally, when interpreting meta-regression outputs, it should be noted that results are only hypothesis generating and do not provide evidence of causality.

## 5 Conclusion

By exploring the relationship between cognition and inflammation in MCI and 10.13039/100020014AD, this study found evidence to support the involvement of sex-specific inflammaging in 10.13039/100020014AD and MCI. Our findings of raised pro-inflammatory markers correlating with worsening cognitive performance provide evidence to support the role of central-peripheral immune crosstalk in the pathogenesis of cognitive impairment in MCI and 10.13039/100020014AD. Inflammation may reduce the integrity of the BBB allowing transfer of cytokines to and from the CNS wherein they might incite microglial and astrocyte inflammatory activity to induce cognitive dysfunction. Further, our findings inform that sex-specific differences may play a role in the inflammatory mechanisms underlying MCI and AD. Females with MCI were found to have increased YKL-40 which may reflect a heightened burden of microglial activity in response to Aβ pathology compared to men, however lower CRP levels may be associated with an impaired capacity for Aβ clearance. These findings were corroborated in investigations of aging, where lower levels of CRP were found in aged individuals. Aging is a key risk factor in AD, and an impaired ability for Aβ clearance with reduced CRP levels may be an important factor underlying this. Furthermore, female AD participants were found to have reduced innate immune activity compared to male subjects, as seen by lower levels of IL1 cytokines. Together these findings may indicate a unique immune response in females along the AD cognitive continuum wherein there is a heightened microglial response in the MCI phase followed by an impaired innate immune response during the AD phase. Additional research exploring this hypothesis is warranted.

## CRediT authorship contribution statement

**Ryan Childs:** Writing – original draft, Software, Project administration, Formal analysis, Data curation. **Diana Karamacoska:** Writing – review & editing, Supervision, Conceptualization. **Chai K. Lim:** Writing – review & editing, Supervision, Software, Formal analysis, Conceptualization. **Genevieve Z. Steiner-Lim:** Writing – review & editing, Supervision, Methodology, Conceptualization.

## Declaration of competing interest

None.

## Data Availability

Data will be made available on request.
