# Supplementary material for: “Let's talk about sex, inflammaging, and cognition, baby”: A meta-analysis and meta-regression of 106 case-control studies on mild cognitive impairment and Alzheimer's disease
Source: Brain Behav Immun Health. 2024 Jul 20;40:100819. doi: 10.1016/j.bbih.2024.100819 (PMC11331696; doi:10.1016/j.bbih.2024.100819)
Supplement: Multimedia component 1 [file mmc1.docx]

Supplementary Table S1. PubMed search strategy.

| Search # | Search terms |
| --- | --- |
| #1 | “cognitive dysfunction”[MeSH] OR “dementia”[MeSH] OR “alzheimer’s disease” OR “mild cognitive impairment” OR AD OR MCI |
| #2 | blood OR serum OR plasma OR urine OR “cerebrospinal fluid” OR CSF OR “plasma”[MeSH] OR “serum”[MeSH] OR “urine”[MeSH] OR “cerebrospinal fluid”[MeSH] |
| #3 | “cytokines”[MeSH] OR inflammation OR cytokines OR chemokine OR interleukin OR interferon OR IL-1β OR IL-2 OR IL-4 OR IL-6 OR IL-8 OR IL-10 OR IL-12 OR IL-18 OR transforming growth factor OR TGF OR tumour necrosis factor OR TNF OR c-reactive protein OR CRP OR interferon OR IFN |
| #4 | “cognition”[MeSH] OR “cognition disorders”[MeSH] OR cognit* OR “executive function” OR perception OR memory OR “decision making” OR language |
| #5 | #1 AND #2 AND #3 AND #4 |

Supplementary Table S2. PsycINFO search strategy.

| Search # | Search terms |
| --- | --- |
| #1 | MA “cognitive dysfunction” OR MA “dementia” OR “alzheimer’s disease” OR “mild cognitive impairment” OR AD OR MCI |
| #2 | blood OR serum OR plasma OR urine OR “cerebrospinal fluid” OR CSF OR MA “plasma” OR MA “serum” OR MA “urine” OR MA “cerebrospinal fluid” |
| #3 | MA “cytokines” OR inflammation OR cytokines OR chemokine OR interleukin OR interferon OR IL-1β OR IL-2 OR IL-4 OR IL-6 OR IL-8 OR IL-10 OR IL-12 OR IL-18 OR transforming growth factor OR TGF OR tumour necrosis factor OR TNF OR c-reactive protein OR CRP OR interferon OR IFN |
| #4 | MA “cognition” OR MA “cognition disorders” OR cognit* OR “executive function” OR perception OR memory OR “decision making” OR language |
| #5 | #1 AND #2 AND #3 AND #4 |

Supplementary Table S3. SCOPUS and Web of Science search strategies

| Search # | Search terms |
| --- | --- |
| #1 | “cognitive dysfunction” OR “dementia” OR “alzheimer’s disease” OR “mild cognitive impairment” OR AD OR MCI |
| #2 | blood OR serum OR plasma OR urine OR “cerebrospinal fluid” OR CSF |
| #3 | inflammation OR cytokines OR chemokine OR interleukin OR interferon OR IL-1β OR IL-2 OR IL-4 OR IL-6 OR IL-8 OR IL-10 OR IL-12 OR IL-18 OR transforming growth factor OR TGF OR tumour necrosis factor OR TNF OR c-reactive protein OR CRP OR interferon OR IFN |
| #4 | cognit* OR “executive function” OR perception OR memory OR “decision making” OR language |
| #5 | #1 AND #2 AND #3 AND #4 |
| #4 | cognit* OR “executive function” OR perception OR memory OR “decision making” OR language |
| #5 | #1 AND #2 AND #3 AND #4 |

Supplementary Table S4. Characteristics of included studies.

| Study | AD Sample Size (n), age (M±SD), female (%) | MCI Sample  Size (n), age (M±SD), female (%) | Control Sample Size (n), age (M±SD), female (%) | Cognitive Measures | Sample | Inflammatory Markers (Method) |
| --- | --- | --- | --- | --- | --- | --- |
| Abe et al. (2020)^1^ | NA | N=296  Age 74.7 ±7.14  Female 35.10% | N=47  Age 75.4 ±5.89  Female 48.90% | MMSE | Serum | CRP, IL3, IL8, IL16, IL13, CXCL10, TNFα (Luminex xMAP) |
| Agrawal et al. (2022)^2^ | N=34  Age 78.3  Female 50% | N=34  Age 78.95  Female 50% | N=34  Age 78.8  Female 50% | MMSE | Plasma | IL21 (ELISA) |
| Alcolea et al. (2014)^3^ | N=59  Age 71  Female 31.8% | N=45  Age 70  Female 48.9% | N=24  Age 64  Female 54.2% | MMSE | CSF | YKL-40 (ELISA) |
| Alsadany et al. (2013)^4^ | N=25  Age 72.2 ±5.9  Female 56.0% | NA | N=25  Age 72.8 ±4.1  Female 52.0% | MMSE | Plasma | IL8 (ELISA) |
| Alvarez et al. (1995)^5^ | N=10  Age 66.6 ±6.4  Female 50.0% | NA | N=20  Age 65.5 ±5.8  Female 55.0% | MMSE | Serum, Plasma | *Serum* IL1β (RIA)  *Plasma* ΤΝFα (ELISA) |
| Amin et al. (2020)^6^ | N=31  Age 73.1±7.4  Female 41.9% | NA | N=13  Age 66.0±8.6  Female 54.8% | MoCA, FCSRT-IR | Serum | IL1β, IL2, IL4, IL6, IL8, IL10, IL12, IL13, TNFα, IFNγ (Multiplex immunoassay) |
| Antonell et al. (2020)^7^ | N=108  Age 63.6 (10.7)  Female 63% | N= 56  Age 67.0 (11.8)  Female 64.3 | N=50  Age 57.0 (17.3)  Female 68% | MMSE | CSF | YKL-40 (ELISA) |
| Araga et al. (1991)^8^ | N=50  Age 78.5±6.4  Female 86.0% | NA | N=37  Age 77.0±7.3  Female 81.1% | MMSE | Serum | IL2, IFNα (ELISA) |
| Baldacci et al. (2017)^9^ | N=35  Age 73(8)  Female 68.6% | N=41  Age 72(10)  Female 34.1% | N=21  Age 64(10)  Female 61.9% | MMSE | CSF | YKL-40 (ELISA) |
| Balldin et al. (2012)^10^ | N=124  Age 76.72±7.8  Female 62.1% | NA | N=196  Age 70.47±8.81  68.4% | MMSE | Serum | IL8, CRP, TNFα (Multiplex fluorescent immunoassay) |
| Berdnarska-Makaruk et al. (2017)^11^ | N=89  Age 72.8±8.13  Female 74.2% | N=113  Age 70.5±8.89  Female 64.6% | N=107  Age 71.3±7.95  Female 64.5% | MMSE | Serum | IL6, hsCRP (ELISA) |
| Björkqvist et al. (2012)^12^ | N=142  Age 76(56-87)  Female 28.2% | N/A | N=174  Age 74(62-99)  Female 67.2% | MMSE | Plasma | RANTES, G-CSF, IL1α, IL3, IL8, IL11, MCP-3, MIP-1, TNFα (ELISA) |
| Blasko et al. (2006)^13^ | N=23  Age 71.9±2.0  Female 65.2% | N/A | N=27  Age 66.9±1.8  Female 51.9% | MMSE | CSF | TNFα, TGFβ, MCP1, MIP1α (ELISA) |
| Boccardi et al. (2019)^14^ | N=129  Age 81.0±6.2  Female 71.3% | N=73  Age 77.5±6.3  Female 61.6% | N=87  Age 75.9±9.0  Female 59.8% | MMSE | Plasma | IFNα2, IFNγ, IL1α, IL1β, IL2, IL5, IL6, IL7, IL10, IL12, IL13, IL15, IL17, TNFα, TNFβ, IL8, IP10, MCP1, MCP1β (ELISA) |
| Boccardi et al. (2023)^15^ | N=40  Age 78.83±3.74  Female 67.5% | N/A | N=40  Age 76.33±3.61  Female 55% | MMSE | Plasma | G-CSF, GM-CSF, INFα, IL3, IL8, IL17 (ELISA) |
| Bonotis et al. (2008)^16^ | *Severe AD*  N=19  Age 75.05±4.9  Female 57.9%  *Mild-moderate AD*  N = 30  Age 76.35±6.9  Female 56.7% | N/A | N=21  Age 71.23±4.4  Female 52.4% | MMSE | Serum | IL1α, IL2, IL6, IL8, IL10 (ELISA) |
| Bossù et al. (2008)^17^ | N=30  Age 71.3±1.6  Female 67% | N/A | N=25  Age 68.5±1.4  Female 56% | MMSE | Serum | IL18 (ELISA) |
| Bozluoclay et al. (2016)^18^ | N=30  Age 73.2±10.2  Female 50.0% | N/A | N=25  Age 73.1±10.6  Female 48% | MMSE | Serum | IL1β, IL6, TNFα, CRP (ELISA) |
| Brosseron et al. (2018)^19^ | N=116  Age 74±8  Female 55% | N=130  Age 21±8  Female 35% | N=85  Age 67±11  Female 35% | MMSE, TMT A, TMT B, FCSRT, semantic fluency, phonetic fluency, word list memory | CSF | IL6, IL8, MCP1, IP10, CRP (Multiplex immunoassay) |
| Bulut et al. (2016)^20^ | N=38  Age 58.7±7.7  Female 68.4% | NA | N=28  Age 58.6±9.0  Female 60.5% | MMSE, AVLT, semantic fluency, lexical fluency, BNT, CDT | Serum | IL1β (ELISA) |
| Callahan et al. (2020)^21^ | N=12  Age 71.37±8.01  Female 66.7% | N=17  Age 76.35±5.82  Female 47.1% | N=20  Age 70.37±8.07  Female 60.0% | RAVLT, MoCA | Serum | TNFα (ELISA) |
| Castañeyra-Ruiz et al. (2016)^22^ | N/A | N=7  Age 77(NR)  Female (NR) | N=8  Age 60 (NR)  Female (NR) | MMSE | CSF | TNFα (ELISA) |
| Cervellati et al. (2018)^23^ | N=110  Age 78(9)  Female 74% | N=258  Age 77(8)  Female 56% | N=75  Age 77(8)  Female 60% | MMSE | Serum | hsCRP (ELISA) |
| Chen et al. (2014)^24^ | N=53  Age 76.7±6.8  Female 69.8% | N/A | N=53  Age 76.7±6.7  Female 69.8% | MMSE | Serum | IL17, IL18, IL23 (ELISA) |
| Cheng et al. (2022)^25^ | N/A | N=180  Age 73.33±5.55  Female 50.0% | N=180  Age 72.78±5.52  Female 50.6% | MoCA | Plasma | IL1β, IL18 (ELISA) |
| Cherbuin et al. (2019)^26^ | N/A | N=81  Age 75.48±1.54  Female NR | N=299  Age 75.31±1.37  Female NR | MMSE | Plasma | IL1β, IL4, IL6, IL8, IL10, TNFα (ELISA) |
| Cisbani et al. (2020)^27^ | N=10  Age 65.7±3.5  Female 50.0% | N=11  Age 70.9±1.6  Female 54.5% | N=11  Age 70.0±2.1  Female 54.5% | MMSE, MoCA | Serum | IL1β, IL4, IL5, IL6, IL10, IL17, TNFα (ELISA) |
| Contreras et al. (2020)^28^ | *APOE4 carriers*  N=18  Age 75.23±7.97  Female 38.89%  *APOE4 noncarriers*  N=15  Age 76.27±11.5  Female 33.33% | N/A | *APOE4 carriers*  N=16  Age 68.05±16.59  Female 43.75%  *APOE4 noncarriers*  N=14  Age 64.88±18.08  Female 50% | MMSE | CSF | TNFα (Multiplex immunoassay) |
| D’Anna et al. (2017)^29^ | N=27  Age 74.26±8.01  Female 51.9% | N/A | N=18  Age 70.67±4.98  Female 44.4% | MMSE | CSF | TNFα (Multiplex immunoassay) |
| Davis et al. (2007)^30^ | N=18  Age NR  Female NR | N/A | N=51  Age NR  Female NR | MMSE | Serum | CRP (Immuno-turbidometric assay) |
| De Kort et al. (2021)^31^ | N=17  Age 67±7  Female 52.9% | N=33  Age 70±3  Female 69.7% | N=50  Age 63±4  Female 50.0% | MMSE | CSF | YKL-40  (ELISA) |
| Demirci et al. (2017)^32^ | N=50  Age 75.80±8.01  Female 62% | N/A | N=30  Age 73.10±5.73  Female 60% | MMSE | Serum | IL18, TNFα (ELISA)  CRP (immuno-nephelometry) |
| Diniz et al. (2010)^33^ | N=31  Female 61.3%  Age 75.8±6.6 | N=72  Female 72.2%  Age 70.5±10.3 | N=64  Female 82.8%  Age 69.1±5.6 | MMSE, CAMCOG, RBMT, FOME, Verbal Fluency, TMT-A, TMT-B, SKT | Serum | TNFα (ELISA) |
| Dukic et al. (2016)^34^ | N=70  Age 74(69-79)  Female 61% | N=48  Age 72(68-76)  Female 65% | N=50  Age 66(63-73)  Female 60% | MMSE, MoCA | Serum | IL6, CRP (Electrochemiluminescence) |
| Dursun et al. (2015)^35^ | N=53  Age 74.02±3.9  Female (NR) | N=30  Age 74.4±2.9  Female (NR) | N=32  Age 72.1±3.4  Female (NR) | MMSE | Serum | IL1α, IL1β, IL6 (ELISA) |
| Edwards et al. (2015)^36^ | N/A | N=73  Age 66.30±8.45  Female 79% | N=211  Age 58.75±6.29  Female 64% | MMSE | Serum | IL5, IL6, IL7, IL10, IL18, TNFα, CRP (electrochemi-luminescence) |
| El-Sayed et al. (2009)^37^ | N=65  Age 71.1±3.2  Female 53.8% | N/A | N=20  Age 69.8±3.1  Female 40% | MMSE | Serum | IL1β (ELISA) |
| Forlenza et al. (2009)^38^ | N=58  Age 76.3±6.5  Female 82.8% | N=74  Age 70.3±10.3  Female 74.3% | N=31  Age 69.9±6.7  Female 29.2% | MMSE, CDT, FOME, RBMT, Trail A and B | Serum | IL1β (ELISA) |
| Fu et al (2021)^39^ | *Moderate and severe*  N=28  Age 79.39±17.13  Female 46.4%  *Mild*  N=30  Age 79.23±10.54  Female 40.0% | N=26  Age 75.2±11.5  Female 28.5% | N=30  Age 73.0±9.2  Female 46.7% | MMSE, MoCA | Serum | IL10, IL35, TGFβ (ELISA) |
| Fu et al. (2022)^40^ | N=20  Age 74.65±1.75  Female 65% | N/A | N=20  Age 73.00±1.71  Female 65% | MMSE | Serum | IL1β, IL6, IL8, IL10, TNFα, hsCRP (ELISA) |
| Galgani et al. (2022)^41^ | N=28  Age 73.1±5.3  Female 57% | N=63  Age 73.0±4.2  Female 62% | N=42  Age 72.3±4.7  Female 60% | MMSE | Plasma | IL1β, IL6, IL10, IL12, TNFα, TGFβ (ELISA) |
| Galimberti et al. (2008)^42^ | N=43  Age 63.3±12.0  Female 72.1% | N/A | N=30  Age 66.0±9.2  Female 66.6% | MMSE | CSF | IL6, IL11 (ELISA) |
| Gangishetti et al. (2018)^43^ | N=32  Age 72.8±7.1  Female 66% | *AD CSF biomarker positive*  N=23  Age 70.6±6.2  Female 56%  *AD CSF biomarker negative*  N=13  Age 70.1±5.0  Female 54% | *AD CSF biomarker positive*  N=13  Age 74.6±6.9  Female 69%  *AD CSF biomarker negative*  N=31  Age 69.1±6.3  Female 61% | MMSE | CSF | IL7, IL10, fractalkine, TNFα, MCP1 (Luminex)  YKL-40  (ELISA) |
| Gezen-Ak et al. (2013)^44^ | N=54  Age 74.22±3.73  Female NR | N=30  Age 74.4±2.9  Female NR | N=32  Age 72.1±3.4  Female NR | MMSE | Serum | IL10, TNFα (ELISA) |
| Giacconi et al. (2022)^45^ | N=95  Age 77.8±0.5  Female 67.7% | N=93  Age 76.1±0.6  Female 63.4% | N=94  Age 72.7  Female 79.6% | MMSE, ADAS-cog | Plasma | IL10, TNFα (ELISA) |
| Gispert et al. (2016)^46^ | N=14  Age 67.1±10.0  Female 73.33% | N=28  Age 70.4±7.2  Female 57.14% | N=53  Age 62.5±7.0  Female 64.15% | MMSE | CSF | YKL-40 (ELISA) |
| Gongora-Rivera et al. (2020)^47^ | N=29  Age 75.50±7.30  Female 82.8% | N/A | N=49  Age 72.85±6.59  Female 75.34% | MMSE | Plasma | IL1α, IL3, IL12, IL16, IL18, IFNα2, MCP3 (LUMINEX assay) |
| Guo et al. (2020)^48^ | N=76  Age 73.34±5.53  Female 38.2% | N/A | N=76  Age 73.11±5.94  Female 40.8% | MMSE, MoCA | Serum | IL1β, IL18 (ELISA) |
| Hao et al. (2021)^49^ | N=200  Age 63.19±9.35  Female 62.5% | N/A | N=174  Age 61.83±9.66  Female 62.6% | MMSE, MoCA, AVLT, BNT, CDT | Serum | CRP (ELISA) |
| Hesse et al. (2016)^50^ | *CSF Donors*  N=41  Age 68(5)  Female 70.7%  *Serum Donors*  N=36  Age 68(2)  Female 56.5% | N/A | *CSF Donors*  N=23  Age 69(10)  Female 56.5%  *Serum Donors*  N=24  Age 70(8)  Female 50.0% | MMSE | CSF, Serum | *CSF* IL1β, IL8, TNFα (electrochemi-luminescence)  *Serum* IL1β, IL8, TNFα (electrochemi-luminescence) |
| Italiani et al. (2018)^51^ | N=60  Age 78.13±8.35  Female 58.3% | N=45  Age 66.98±8.14  Female 57.8% | N=94  Age 68.64±6.95  Female 56.4% | MMSE | Serum | IL1α, ΙL1β, IL18, IL33 (ELISA) |
| Ju et al. (2022)^52^ | N=20  Age 70.75±7.32  Female 55% | N=22  Age 66.91±5.36  Female 54.5% | N=22  Age 68.73±6.79  Female 40.9% | MMSE | Serum | IL17, TNFα (ELISA) |
| Kalman et al. (1997)^53^ | *Severe*  N=26  Age 75.4±6.43  Female 65.4%  *Mild-moderate*  N=15  Age 70.1±5.19  Female 60.0% | N/A | N=24  Age 72.9±4.27  Female 75.0% | MMSE | CSF | IL6 (ELISA) |
| Kamer et al. (2009)^54^ | N=18  Age NR  Female 78% | N/A | N=16  Age NR  Female 94% | MMSE | Plasma | IL1β, IL6, TNFα (Multiplex) |
| Kassner et al. (2008)^55^ | N=5  Age 76 (65-79)  Female 60% | N/A | N=5  Age 61 (62-81)  Female 60% | MMSE | Plasma | TNFα (ELISA) |
| Khedr et al. (2020)^56^ | N=15  Age 65.7±5.1  Female 60% | N/A | N=25  Age 60.7±6.8  Female 48% | 3MS, MAS | Serum | TGFβ1 (ELISA) |
| Kim et al. (2011)^57^ | N=18  Age 75.9±6.0  Female 50.0% | N=20  Age 76.1±2.8  Female 55.0% | N=21  Age 75.5±1.3  Female 52.4% | MMSE | Plasma | IL8, IL10, MCP1, TNFα (ELISA) |
| King et al. (2019)^58^ | N=20  Age 75.9±6.7  Female 25% | N=21  Age 78.5±6.4  Female 66.7% | N=20  Age 75.9±7.3  Female 20% | MMSE | Serum | IL1β, IL2, IL4, IL6, IL8, IL10, IL13, TNFα (ELISA)  CRP (electrochemi-luminescence) |
| Koutentaki et al. (2023)^59^ | N/A | N=99  Age 75.3±6.4  Female 74.7% | N=84  Age 72.7±7.4  Female 65.5% | MMSE | Plasma | IL6 (ELISA) |
| Lawlor et al. (1996)^60^ | N=17  Age 74.94±5.34  Female 70.59 | N/A | N=15  Age 68.47±6.84  Female 80% | MMSE | Serum | CRP (Nephelometry) |
| Le Page et al. (2017)^61^ | N=15  Age 78.1±4.37  Female 80% | N=13  Age 72.8±3.64  Female 70% | N=13  Age 71.1±5.22  Female 77% | MMSE, MoCA | Serum | IL1β, IL6, IL10, TNFα, IFNγ, IP10 (Luminex) |
| Liang et al. (2020)^62^ | N=39  Age 77.7±8.5  Female 74.4% | N=35  Age 75.6±8.4  Female 65.7% | N=26  Age 68.7±4.2  Female 80.8% | MMSE, Verbal learning test, Digit span, TMT-A | Plasma | IL1β, IL33 (Multiplex) |
| Liang et al. (2021)^63^ | N=28  Age 78.3±8.8  Female 75% | N=51  Age 75.6±8.6  Female 78.4% | N=12  Age 66.3±5.9  75% | MMSE, Verbal learning test, TMT-A, BNT | Plasma | IL2, IFNγ, TNFα, IL4, IL5, IL6, IL10, IL13, IL1β, IL23, IL25, IL31, IL17, IL8, IP10, MCP1, MIP1α, MIP1β, RANTES, eotaxin, IL7, IL9, G-CSF, GM-CSF (Multiplex) |
| Llano et al. (2012)^64^ | N=15  Age 70.2±7.4  Female 20.0% | N/A | N=7  Age 65.0±5.2  Female 28.6% | MMSE | CSF, plasma | IFNγ, IL1β, IL2, IL6, IL8, IL10, TNFα (ELISA) |
| Lleo et al. (2019)^65^ | N=110  Age 68.5±8.5  Female 42.7% | N=128  Age 67±8.4  Female 38.3% | N=154  Age 58.2±7.2  Female 55.8% | MMSE | CSF | YKL-40 (ELISA) |
| Lourenco et al. (2021)^66^ | N=14  Age 74.2±7.1  Female 71.4% | N=14  Age 71.6±5.9  Female 42.9% | N=25  Age 67.8±4.8  Female 60.0% | MMSE | CSF | IFNα, IFNγ, IL1β, IL2, IL4, IL5, IL6, IL7, IL8, IL10, IL13, IL15, IL17, IP10, MCP1, MIP1α, MIP1β, TNFα (ELISA) |
| Lu et al. (2020)^67^ | N=187  Age 75.2±6.1  Female 46% | N=100  Age 75.3±5.7  Female 40% | N=80  Age 74.9±5.4  Female 40% | MMSE | Serum | CRP (ELISA) |
| Marksteiner et al. (2011)^68^ | N=96  Age 77.0±0.8  Female NR | N=44  Age 73.5±1.2  Female NR | N=19  Age 72.1±1.3  Female NR | MMSE | Plasma | G-CSF, IL1α, IL3, IL8, IL11, MCP-3, M-CSF, RANTES, TNFα (protein microarray) |
| Malaguarnera et al. (2006)^69^ | N=40  Age 73.2±7.08  Female 60% | N/A | N=30  Age 73.5±3.24  Female 50% | MMSE | Plasma | IL18, TGFβ (ELISA) |
| Motta et al. (2007)^70^ | *Moderate*  N=22  Age 73.1±5.52  Female 54.5%  *Mild*  N=18  Age 71±4.12  61.1% | N/A | N=20  Age 72.5±2.38  Female 50% | MMSE | Plasma | IL12, IL16, IL18, TGFβ (ELISA) |
| Muszyński et al. (2017)^71^ | N=45  Age NR  Female 80.0% | N=18  Age NR  Female 55.6% | N=23  Age NR  Female 65.2% | MMSE | CSF | YKL-40 (ELISA) |
| Nie et al. (2022)^72^ | N/A | N=23  Age 73.0±4.5  Female 56.5% | N=23  Age 72.5±0.8  Female 60.9% | MoCA, WMDS, COWAT, CFT | Plasma | IL1βb, IL2, IL4, IL6, TNFα, IFNγ, Eotaxin, MIP3α, MCP-1, MCP-3, MCP-4, MIP1α (Luminex) |
| Nordengen et al. (2019)^73^ | N=27  Age 67.6±5.2  Female 48% | N=40  Age 66.6±7.4  Female 57% | N=36  Age 61.1±9.2  Female 53% | MMSE | CSF | MCP-1, YKL40, Fractalkine (ELISA) |
| Park et al. (2021)^74^ | N=26  Age 75.54±6.17  Female 69.2% | N=28  Age 75.60±6.15  Female 50.0% | N=25  Age 75.56±6.29  Female 44.0% | MMSE | Blood | IL1β, TGFβ, CRP (NR) |
| Petersen et al. (2020)^75^ | N/A | N=85  Age 66.3±8.3  Female 65% | N=378  Age 58.9±6.5  Female 81% | TMT-B, Logical memory, Visual reproduction, BNT, COWAT | Serum | IL1β, IL5, IL6, IL7, IL10, IL18, TNFα, CRP (ELISA) |
| Porcellini et al. (2008)^76^ | N=142  Age 77±8  Female 66.2% | N/A | N=830  Age 73±6  Female 54.2% | MMSE | Plasma | CRP (Immuno-ephelometricy) |
| Reale et al. (2012)^77^ | N=38  Age 73.8±5.5  Female 47.4% | N/A | N=39  Age 72.7±4.8  Female 53.8% | MMSE, ADAS-Cog | Plasma | IL18, MCP-1, RANTES (ELISA) |
| Rizzi & Roriz-Cruz (2017)^78^ | N/A | N=33  Age 68(61-78)  Female 68.7% | N=12  Age 63.5(60-76)  Female 75% | CERAD, CDT | CSF | IL6, TNFα (ELISA)  IL1β (enzyme amplified sensitivity immunoassay) |
| Rota et al. (2006)^79^ | N=30  Age 71±6.03  Female 60% | N/A | N=25  Age 69±8.6  Female 40% | MMSE | CSF, Serum | IL12, IL10, TGFβ, IFNγ (ELISA) |
| Rui et al. (2021)^80^ | N=33  Age 65.66±9.22  Female 69.7% | N=33  Age 63.61±8.88  Female 69.7% | N=20  Age 59.90±6.18  Female 55.0% | MMSE, MoCA | Plasma, CSF | IL1β (ELISA) |
| Savaş et al. (2019)^81^ | N=46  Age 76.±9  Female 67.4% | N/A | N=30  Age 72±9  Female 60% | MMSE | Serum | TNFα (ELISA) |
| Shen et al. (2019)^82^ | N/A | N=57  Age 68.77±5.47  Female 31.6% | N=57  Age 67.77±5.16  Female 21.6% | SM-MMSE, MoCA | Plasma | TNFα, IP10, IL8, MCP1, IL4 (LUMINEX assay)  hsCRP, hsIL6 (ELISA) |
| Shen et al. (2021)^83^ | N=60  Age 63.4±8.4  Female 58.3% | N/A | N=50  Age 60.7±8.8  Female 52% | MMSE | CSF | IL1β, IL6 (ELISA) |
| Smith et al. (2011)^84^ | N=34  Age 77.1±6.8  Female 50% | N/A | N=34  Age 76.6±6  Female 50% | MMSE | Plasma | IL6, TNFα (ELISA) |
| Song et al. (2015)^85^ | N=532  Age 75.86±7.94  Female 62.2% | N/A | N=319  Age 74.47±9.4  Female 75.1% | MMSE | Serum | hsCRP (NR) |
| Startin et al. (2019)^86^ | N=27  Age 59.33±4.04  Female 33.3% | N/A | N=27  Age 49.26±10.40  Female 40.7% | MMSE | Plasma | IL1β, IL6, IL10, TNFα (Bead-based immunoassay) |
| Sun et al. (2016)^87^ | N=40  Age 79.96±6.93  Female 57.5% | N/A | N=40  Age 77.89±6.93  Female 50.0% | MMSE | Serum | IL1β, IL6, TNFα, hsCRP (ELISA) |
| Sun et al. (2022)^88^ | N=30  Age 75.27±7.67  Female 40% | N/A | N=44  Age 71.86±7.76  Female 50% | MMSE, DSR, CDT, TMT-A, TMT-B | Plasma | IL1β, IL6, IL8, TNFα (Bead-based Immunoassay) |
| Teunissen et al. (2003)^89^ | N=34  Age 73 (53-95)  Female 59% | N/A | N=61  Age 68 (55-86)  Female 43% | MMSE, Word learning test, Delayed recall, DCT, Stroop coloured word test | Serum | IL6, CRP (ELISA) |
| Tisato et al. (2016)^90^ | N=168  Age 79 (75-83)  Female 73% | N=251  Age 77 (73-80)  Female 59% | N=56  Age 74 (67-79)  Female 68% | MMSE | Serum | hsCRP (Enzymatic colorimetry) |
| Villarreal et al. (2016)^91^ | N=28  Age 81.9±9.2  Female 78.6% | N=30  Age 81.2±7.8  Female 66.7% | N=77  Age 76.5±6.7  Female 64.9% | MMSE, CDT | Serum | IL1β, IL5, IL6, IL7, IL10, IL18, TNFα, CRP, MIP1α (ELISA) |
| Vurgen et al. (2013)^92^ | N=40  Age NR  Female 70% | N/A | N=40  Age NR  Female 67.5% | MMSE, RAVLT, Benton facial recognition test, category fluency | Serum | CRP (Nephelometry) |
| Walker et al. (2020)^93^ | N/A | N=26  Age 66.3±9.7  Female 58% | N=150  Age 64.3±8.6  Female 63% | MMSE | Plasma | IL6 (ELISA) |
| Wang et al. (2020)^94^ | N=11  Age 73.6±5.6  Female 63.6% | N=63  Age 73.8±6.4  Female 28.6% | N=35  Age 75.9±5.2  Female 57.1% | MMSE, AVLT, TMT-A/B, BNT | CSF | YKL-40 (ELISA) |
| Wang et al. (2014)^95^ | N=97  Age 73.7±9.4  Female 44.3% | N=54  Age 76.6±9.1  Female 57.4% | N=122  Age 73.7±8.4  Female 54.1% | MMSE | Plasma | IL6, IL10 (ELISA) |
| Wennberg et al. (2019)^96^ | N/A | N=186  Age 79.7 (73.1-84.7)  Female 40.9% | N=1,416  Age 71.9 (63.6-78.4)  Female 47.3% | AVLT, WMS, BNT, TMT-B, Category fluency | Plasma | IL6, IL10, TNFα (Bead-based immunoassay) |
| Wennström et al. (2015) [a]^97^ | N=45  Age 76(61-86)  Female 71.1% | N/A | N=36  Age 64(44-82)  Female 55.6% | MMSE | CSF | IL1β, IL6, TNFα, IFNγ (ELISA) |
| Wennström et al. (2015) [b] ^98^ | N=49  Age 77.1±6.0  Female 76% | N/A | N=44  Age 63.7±10.3  Female 52% | MMSE | CSF | YKL-40 (ELISA)  IL8, IFNγ, IP10, MCP1, MCP4, MIP1β (ELISA) |
| Wennström et al. (2014)^99^ | N=19  Age 75(72-79)  Female 52.6% | N/A | N=20  Age 76(71-84)  Female 50.0% | MMSE | CSF | MCP1α (ELISA) |
| Xin et al. (2021)^100^ | N=80  Age 65.7±5.4  Female 35.00% | N/A | N=71  Age 66.2±5.8  Female 38.03% | MMSE | Serum | IL1β, IL6, CRP (ELISA) |
| Xu et al. (2020)^101^ | N=97  Age 72.29±8.67  Female 47.4% | N=50  Age 72.08±9.61  Female 40.0% | N=84  Age 72.08±9.61  Female 54.8% | MMSE, MoCA | Urine | MCP1 (ELISA) |
| Yamamoto et al. (2005)^102^ | N=61  Age 80±6  Female 61% | N/A | N=32  Age 77±5  Female 47% | MMSE | Plasma | Hs-CRP (Immunonephelometric assays) |
| Yarchoan et al. (2013)^103^ | N=203  Age 74.5±7.7  Female 58% | N=58  Age 71.6±8.4  Female 52% | N=117  Age 69.9±10.1  Female 68% | MMSE | Plasma | CRP (ELISA) |
| Yeram et al. (2021)^104^ | N=71  Age 70±7.02  Female 35.71% | N/A | N=70  Age 68.65±5.98  Female 42.86% | MMSE | Serum | IL6 (chemi-luminescence immunoassay) |
| Yu et al. (2017)^105^ | N=156  Age 72.69±10.15  Female 51.92% | N/A | N=156  Age 74.40±9.15  Female 40.38% | MMSE | Serum | IL1α, IL3, IL5, IL13, IL15, IFNγ, TNFα, MCP1, MCP3, MIP1β, IP10, Fractalkine (chemi-luminescence LUMINEX) |
| Zhao et al. (2012)^106^ | N/A | N=150  Age 70.67±4.27  Female 47.8% | N=150  Age 69.85±5.21  Female 41.3% | MMSE, MoCA, AVLT | Serum | IL6, IL10, TNFα, IFNγ (ELISA) |
| Zhou et al. (2020)^107^ | N=60  Age 68.6±9.1  Female 45.0% | N/A | N=60  Age 68.3±8.3  Female 53.3% | MMSE | Serum | CRP (immuno-turbidometric assay) |
| Zorkina et al. (2023)^108^ | N/A | N=142  Age 72.5±7.4  Female 100% | N=75  Age 68.9±6.2  Female 100% | MMSE, MoCA | Serum | IL1α, IL4, IL6, IL7, IL8, IP-10, MCP-1, MIP-1β, TNFα, CRP (Luminex Immunoassay) |
| Zuliana et al. (2007)^109^ | N=60  Age 78.5±7.6  Female 65% | N/A | N=42  Age 72.5±5.8  Female 46% | MMSE | Plasma, Serum | *Plasma* IL1β, IL6, TNFα (ELISA)  *Serum* IL10 (ELISA) |
| Zuliani et al. (2008)^110^ | N=60  Age 78.5±7.6  Female 65% | N/A | N=30  Age 74.5±5.1  Female 48% | MMSE | Plasma | IL1β, IL6, TNFα, IFNγ  (ELISA) |
| Zuliani et al. (2023)^111^ | N=176  Age 80±5  Female 70% | N=166  Age 78±5  Female 54% | N=151  Age 75±6  Female 53% | MMSE, Rey’s 15 words, Raven progressive test, CDT | Serum | hsCRP (ELISA) |

AD = Alzheimer’s Disease, MCI = mild cognitive impairment, CSF = cerebrospinal fluid, IL = Interleukin, MMSE = mini mental state exam, M+SD = mean and standard deviation, NA = not available, FCSRT = free and cued selective recall test, MoCA = Montreal cognitive assessment, TMT = trail making test, AVLT = auditory verbal learning test, BNT = Boston naming test, CDT = clock drawing test, CAMCOG = Cambridge cognitive examination, RBMT = Rivermead Behavioural Memory Test, FOME = Fuld objective memory Evaluation, SKT = short cognitive performance test, ADAS-Cog = Alzheimer’s disease assessment scale cognitive subscale, WMDS = Weschler’s memory digit span, COWAT = controlled oral word association test, CFT = categorial verbal fluency test, DRS = dementia rating scale, DCT = dot counting test.

Supplementary Table S5. Meta-analysis results.

| AD Serum and Plasma | | | | | | | | | |
| --- | --- | --- | --- | --- | --- | --- | --- | --- | --- |
| Marker | Studies | N | HG (95% CI) | Z | P | Q (dF) | Tau2 | I2 | Egger’s regression 2-tailed *p*-value |
| IL1a | 6 | AD = 433  HC = 418 | 0.064 (-0.360 – 0.488) | 0.297 | 0.766 | 39.448 (5) | 0.228 | 87.325 | 0.126 |
| IL1β | 25 | AD = 1012  HC = 937 | 0.671 (0.258 – 1.083) | 3.188 | 0.001 | 418.039 (24) | 1.045 | 94.259 | 0.017 |
| IL2 | 8 | AD = 376  HC = 302 | 0.412 (-0.106 – 0.930) | 1.557 | 0.119 | 60.479 (7) | 0.471 | 88.426 | 0.341 |
| IL4 | 3 | AD = 79  HC = 63 | 0.027 (-0.450 – 0.503) | 0.109 | 0.913 | 3.951 (2) | 0.088 | 49.376 | 0.697 |
| IL5 | 4 | AD = 222  HC = 256 | 0.112 (-0.114 – 0.338) | 0.969 | 0.332 | 3.517 (3) | 0.098 | 14.70 | 0.675 |
| IL6 | 25 | AD = 1103  HC = 1095 | 0.381 (0.092 – 0.670) | 2.583 | 0.010 | 244.848 (24) | 0.469 | 90.198 | 0.0572 |
| IL7 | 3 | AD = 212  HC = 245 | -0.33 (-0.350 – 0.284) | -0.204 | 0.838 | 3.705 (2) | 0.037 | 46.021 | 0.815 |
| IL8 | 10 | AD = 384  HC = 407 | 0.798 (0.013 – 1.583) | 1.992 | 0.046 | 199.247 (9) | 1.491 | 95.483 | 0.617 |
| IL10 | 18 | AD = 903  HC = 951 | 0.318 (-0.028 – 0.664) | 1.801 | 0.072 | 200.37 (17) | 0.485 | 91.514 | 0.461 |
| IL12 | 5 | AD = 156  HC = 132 | 0.338 (-0.269 – 0.944) | 1.091 | 0.275 | 23.754 (4) | 0.389 | 83.161 | 0.567 |
| IL17 | 5 | AD = 110  HC = 104 | 0.543 (-0.588 – 1.673) | 0.941 | 0.347 | 77.464 (4) | 1.552 | 94.836 | 0.532 |
| IL18 | 10 | AD = 455  HC = 493 | 1.180 (0.322 – 2.039) | 2.693 | 0.007 | 298.710 (9) | 1.851 | 96.987 | 0.019 |
| IFNγ | 7 | AD = 320  HC = 267 | 0.153 (-0.192 – 0.498) | 0.869 | 0.385 | 18.092 (6) | 0.130 | 66.835 | 0.097 |
| CRP | 13 | AD = 1107  HC = 1705 | 0.355 (0.037 – 0.674) | 2.186 | 0.029 | 147.715 (12) | 0.277 | 91.876 | 0.074 |
| hsCRP | 8 | AD = 1095  HC = 745 | 0.649 (0.284 – 1.013) | 3.483 | <0.001 | 72.877 (7) | 0.228 | 90.395 | 0.234 |
| TNFα | 28 | AD = 1103  HC = 1149 | 0.392 (-0.018 – 0.801) | 1.873 | 0.061 | 532.679 (27) | 1.125 | 94.931 | 0.052 |
| TGFβ | 6 | AD = 222  HC = 172 | 0.649 (-0.571 – 1.868) | 1.042 | 0.297 | 143.112 (5) | 2.233 | 96.506 | 0.146 |
| ΜCP-1 | 3 | AD = 222  HC = 207 | -6.924 (-14.419 – 0.570) | -1.811 | 0.070 | 605.946 (2) | 43.563 | 99.670 | 0.296 |
| AD CSF | | | | | | | | | |
| IL6 | 6 | AD = 320  HC = 232 | 0.892 (-0.178 – 1.962) | 1.634 | 0.102 | 148.723 (5) | 1.701 | 96.638 | 0.315 |
| IL8 | 4 | AD = 186  HC = 140 | -0.139 (-0.518 – 0.239) | -0.720 | 0.471 | 6.191 (3) | 0.074 | 51.542 | 0.421 |
| MCP-1 | 5 | AD = 199  HC = 220 | 0.793 (0.168 – 1.417) | 2.489 | 0.013 | 30.263 (4) | 0.427 | 86.782 | 0.0378 |
| YKL40 | 7 | AD = 371  HC = 388 | 1.155 (0.872 – 1.438) | 8.010 | <0.0001 | 15.723 (6) | 0.085 | 61.840 | 0.991 |
| MCI Serum and Plasma | | | | | | | | | |
| IL1α | 4 | MCI = 271  HC = 267 | -0.071 (-0.299 – 0.158) | -0.604 | 0.546 | 5.574 (3) | 0.018 | 34.416 | 0.039 |
| IL1β | 11 | MCI = 519  HC = 1013 | 0.344 (-0.060 – 0.748) | 1.669 | 0.095 | 103.978 (10) | 0.402 | 90.383 | 0.116 |
| IL2 | 3 | MCI = 95  HC = 55 | 1.569 (-0.404 – 3.541) | 1.559 | 0.119 | 44.635 (2) | 2.873 | 95.519 | 0.051 |
| IL4 | 6 | MCI = 375  HC = 486 | 0.481 (-0.238 – 1.200) | 1.311 | 0.190 | 89.264 (5) | 0.713 | 94.399 | 0.101 |
| IL5 | 5 | MCI = 250  HC = 689 | 0.026 (-0.149 – 0.201) | 0.295 | 0.768 | 4.632 (4) | 0.006 | 13.638 | 0.841 |
| IL6 | 17 | MCI = 1256  HC = 2851 | 0.033 (-0.119 – 0.185) | 0.430 | 0.667 | 52.614 (16) | 0.061 | 69.590 | 0.631 |
| IL7 | 5 | MCI = 381  HC = 753 | -0.135 (-0.272 – 0.002) | -1.932 | 0.053 | 2.142 (4) | 0.000 | 0.000 | 0.333 |
| IL8 | 6 | MCI = 614  HC = 476 | -0.134 (-0.482 – 0.213) | -0.759 | 0.448 | 24.150 (5) | 0.138 | 79.296 | 0.886 |
| IL10 | 13 | MCI = 916  HC = 2787 | 0.143 (-0.029 – 0.314) | 1.630 | 0.103 | 42.341 (12) | 0.062 | 71.659 | 0.394 |
| IL18 | 5 | MCI = 529  HC = 807 | 0.197 (-0.015 – 0.408) | 1.824 | 0.068 | 9.442 (4) | 0.033 | 57.636 | 0.180 |
| MCP-1 | 5 | MCI = 346  HC = 254 | -0.180 (-0.904 – 0.544) | -0.488 | 0.626 | 65.922 (4) | 0.630 | 93.932 | 0.675 |
| TNFα | 17 | MCI = 1323  HC = 2868 | 0.030 (-0.472 – 0.531) | 0.116 | 0.908 | 589.375 (16) | 1.056 | 97.285 | 0.251 |
| IFNγ | 4 | MCI = 106  HC = 66 | 0.425 (-0.220 – 1.071) | 1.291 | 0.197 | 11.699 (3) | 0.320 | 74.357 | 0.962 |
| CRP | 7 | MCI = 734  HC = 829 | -0.025 (-0.226 – 0.176) | -0.242 | 0.808 | 18.152 (6) | 0.047 | 66.947 | 0.890 |
| hsCRP | 5 | MCI = 733  HC = 542 | 0.014 (-0.232 – 0.260) | 0.111 | 0.912 | 14.047 (4) | 0.055 | 71.524 | 0.485 |
| MCI CSF | | | | | | | | | |
| MCP-1 | 3 | MCI = 184  HC = 173 | 0.328 (-0.706 – 1.362) | 0.621 | 0.534 | 32.948 (2) | 0.775 | 93.930 | 0.343 |
| YKL40 | 7 | MCI = 371  HC = 388 | 0.950 (0.584 – 1.316) | 5.085 | <0.0001 | 30.913 (6) | 0.191 | 80.591 | 0.066 |

AD = Alzheimer’s disease, MCI = mild cognitive impairment, HC = healthy control, CSF = cerebrospinal fluid, CRP = C-reactive protein, TNF = tumour necrosis factor, IFN = interferon, dF = degrees of freedom, IL = interleukin, HG = Hedges’ G, MCP-1 = monocyte chemoattractant protein 1, YKL-40 = chitinase-3-like protein 1.

Supplementary material Table S6. Function of identified inflammatory markers, both peripherally and centrally.

| **Marker** | **Peripheral** | **Central** |
| --- | --- | --- |
| IL1α | Present in almost all haematopoietic and non-haematopoietic cells, particularly endothelial barrier cells, and acts as a generator of acute inflammation by recruiting neutrophils and upregulating further pro-inflammatory cytokine production ^112^ | Stimulates microglial pro-inflammatory activity after injury ^113^. |
| IL4 | Anti-inflammatory cytokine with roles in both the innate and adaptive immune systems, with functions including plasma cell differentiation and modulation T cell cytokine production ^114^. | Increase amyloid-β degradation and uptake by microglia through autophagy ^115^. |
| IL6 | Pro-inflammatory cytokine with innate immunity (released by monocytes and macrophages, recruits neutrophils in acute inflammation and monocytes in chronic inflammation) and adaptive immune activity (plasma cell and T helper 17 cell differentiation) ^116^. | Mediates the transition from innate to adaptive immunity in the CNS. Induces a microglial pro-inflammatory state and induces reactive astrogliosis ^117,118^. |
| IL8 | A chemokine released in acute inflammation by a number of cells including monocytes, lymohpcytes, and granulocytes. Recruits and activates neutrophils to release granule contents ^119^. | Released by astrocytes and microglia and promotes chronic neuroinflammation and oxidative stress ^120^. |
| IL18 | An IL1 family pro-inflammatory cytokine which stimulates natural killer cells, macrophages, and T-cell differentiation ^121^. | Stimulates release of pro-inflammatory cytokines from microglia and astrocytes and contributes to oxidative stress and amyloidogenesis ^122^. |
| CRP | Released in acute phase inflammation with a primary role in activating the complement system, as well as promoting apoptosis and upregulation of cytokines ^123^ | Induces localised microglial activation surrounding Aβ plaques ^124^. |
| TNFα | Released by antigen presenting cells and T cells. Regulates effector T cell and NKT cell activity and communication with other immune cells ^125^. | Released in large quantities by microglia with NFκΒ activation, and in turn is a potent activator of the NFκB pathway and oxidative stress ^126,127^. |
| MCP-1 | A chemokine released primarily by monocytes and macrophages which regulates migration of helper T cells, NKT cells, and monocytes ^128^. | Released primarily by astrocytes in response to Aβ and induces microglial proliferation ^129^. |
| YKL-40 | Released by macrophages, fibroblast-like synovial cells, and chrondrocytes with roles in tissue remodelling and angiogenesis ^130^. | A chitin-binding lectin released in neuroinflammatory states by astrocytes and is involved in microglial activation and the remodelling of the extracellular matrix ^131,132^. |

References

1. Abe K, Chiba Y, Hattori S, et al. Influence of plasma cytokine levels on the conversion risk from MCI to dementia in the Alzheimer’s disease neuroimaging initiative database. *Journal of the Neurological Sciences*. 2020;414:116829. doi:10.1016/j.jns.2020.116829

2. Agrawal S, Baulch JE, Madan S, et al. Impact of IL-21-associated peripheral and brain crosstalk on the Alzheimer’s disease neuropathology. *Cell Mol Life Sci*. 2022;79(6):331. doi:10.1007/s00018-022-04347-6

3. Alcolea D, Carmona-Iragui M, Suárez-Calvet M, et al. Relationship Between β-Secretase, Inflammation and Core Cerebrospinal Fluid Biomarkers for Alzheimer’s Disease. *JAD*. 2014;42(1):157-167. doi:10.3233/JAD-140240

4. Alsadany MA, Shehata HH, Mohamad MI, Mahfouz RG. Histone Deacetylases Enzyme, Copper, and IL-8 Levels in Patients With Alzheimer’s Disease. *Am J Alzheimers Dis Other Demen*. 2013;28(1):54-61. doi:10.1177/1533317512467680

5. alvarez X. Blood Levels of Histamine, IL-1 , and TNF-c in Patients with Mild to Moderate Alzheimer Disease. *tumor necrosis factor*. 1996;29:16.

6. Amin J, Boche D, Clough Z, et al. Peripheral immunophenotype in dementia with Lewy bodies and Alzheimer’s disease: an observational clinical study. *J Neurol Neurosurg Psychiatry*. 2020;91(11):1219-1226. doi:10.1136/jnnp-2020-323603

7. Antonell A, Tort‐Merino A, Ríos J, et al. Synaptic, axonal damage and inflammatory cerebrospinal fluid biomarkers in neurodegenerative dementias. *Alzheimer’s &amp; Dementia*. 2020;16(2):262-272. doi:10.1016/j.jalz.2019.09.001

8. Araga S, Kagimoto H, Funamoto K, Takahashi K. Reduced natural killer cell activity in patients with dementia of the Alzheimer type. *Acta Neurologica Scandinavica*. 1991;84(3):259-263. doi:10.1111/j.1600-0404.1991.tb04948.x

9. Baldacci F, Toschi N, Lista S, et al. Two‐level diagnostic classification using cerebrospinal fluid YKL‐40 in Alzheimer’s disease. *Alzheimer’s &amp; Dementia*. 2017;13(9):993-1003. doi:10.1016/j.jalz.2017.01.021

10. Balldin VH, Hall JR, Barber RC, Hynan L, Diaz-Arrastia R, O’Bryant SE. The Relation between Inflammation and Neuropsychological Test Performance. *International Journal of Alzheimer’s Disease*. 2012;2012:1-6. doi:10.1155/2012/703871

11. Bednarska-Makaruk M, Graban A, Wiśniewska A, et al. Association of adiponectin, leptin and resistin with inflammatory markers and obesity in dementia. *Biogerontology*. 2017;18(4):561-580. doi:10.1007/s10522-017-9701-0

12. Björkqvist M, Ohlsson M, Minthon L, Hansson O. Evaluation of a Previously Suggested Plasma Biomarker Panel to Identify Alzheimer’s Disease. Block ML, ed. *PLoS ONE*. 2012;7(1):e29868. doi:10.1371/journal.pone.0029868

13. Blasko I, Knaus G, Weiss E, et al. Cognitive deterioration in Alzheimer’s disease is accompanied by increase of plasma neopterin. *Journal of Psychiatric Research*. 2007;41(8):694-701. doi:10.1016/j.jpsychires.2006.02.001

14. Boccardi V, Paolacci L, Remondini D, et al. Cognitive Decline and Alzheimer’s Disease in Old Age: A Sex-Specific Cytokinome Signature. Polidori MC, ed. *JAD*. 2019;72(3):911-918. doi:10.3233/JAD-190480

15. Boccardi V, Poli G, Cecchetti R, et al. miRNAs and Alzheimer’s Disease: Exploring the Role of Inflammation and Vitamin E in an Old-Age Population. *Nutrients*. 2023;15(3):634. doi:10.3390/nu15030634

16. Bonotis K, Krikki E, Holeva V, Aggouridaki C, Costa V, Baloyannis S. Systemic immune aberrations in Alzheimer’s disease patients. *Journal of Neuroimmunology*. 2008;193(1-2):183-187. doi:10.1016/j.jneuroim.2007.10.020

17. Bossù P, Ciaramella A, Salani F, et al. Interleukin-18 produced by peripheral blood cells is increased in Alzheimer’s disease and correlates with cognitive impairment. *Brain, Behavior, and Immunity*. 2008;22(4):487-492. doi:10.1016/j.bbi.2007.10.001

18. Bozluolcay M, Andican G, Fırtına S, Erkol G, Konukoglu D. Inflammatory hypothesis as a link between Alzheimer’s disease and diabetes mellitus: AD and DM. *Geriatr Gerontol Int*. 2016;16(10):1161-1166. doi:10.1111/ggi.12602

19. Brosseron F, Traschütz A, Widmann CN, et al. Characterization and clinical use of inflammatory cerebrospinal fluid protein markers in Alzheimer’s disease. *Alz Res Therapy*. 2018;10(1):25. doi:10.1186/s13195-018-0353-3

20. Bulut O, Tanburoğlu A, Bölük G, et al. Examination of IL-1β level as an inflammasome marker in Alzheimer’s disease. :7.

21. Callahan CM, Apostolova LG, Gao S, et al. Novel Markers of Angiogenesis in the Setting of Cognitive Impairment and Dementia. De La Torre J, ed. *JAD*. 2020;75(3):959-969. doi:10.3233/JAD-191293

22. Castañeyra-Ruiz L, González-Marrero I, Carmona-Calero EM, et al. Cerebrospinal fluid levels of tumor necrosis factor alpha and aquaporin 1 in patients with mild cognitive impairment and idiopathic normal pressure hydrocephalus. *Clinical Neurology and Neurosurgery*. 2016;146:76-81. doi:10.1016/j.clineuro.2016.04.025

23. Cervellati C, Trentini A, Bosi C, et al. Low-grade systemic inflammation is associated with functional disability in elderly people affected by dementia. *GeroScience*. 2018;40(1):61-69. doi:10.1007/s11357-018-0010-6

24. Chen JM, Jiang GX, Li QW, Zhou ZM, Cheng Q. Increased Serum Levels of Interleukin-18, -23 and -17 in Chinese Patients with Alzheimer’s Disease. *Dement Geriatr Cogn Disord*. 2014;38(5-6):321-329. doi:10.1159/000360606

25. Cheng L, Dong R, Song C, et al. Mediation Effects of IL-1β and IL-18 on the Association Between Vitamin D Levels and Mild Cognitive Impairment Among Chinese Older Adults: A Case–Control Study in Taiyuan, China. *Front Aging Neurosci*. 2022;14:836311. doi:10.3389/fnagi.2022.836311

26. Cherbuin N, Walsh E, Baune BT, Anstey KJ. Oxidative stress, inflammation and risk of neurodegeneration in a population sample. *Eur J Neurol*. 2019;26(11):1347-1354. doi:10.1111/ene.13985

27. Cisbani G, Koppel A, Knezevic D, Suridjan I, Mizrahi R, Bazinet RP. Peripheral cytokine and fatty acid associations with neuroinflammation in AD and aMCI patients: An exploratory study. *Brain, Behavior, and Immunity*. 2020;87:679-688. doi:10.1016/j.bbi.2020.02.014

28. Contreras JA, Aslanyan V, Sweeney MD, et al. Functional connectivity among brain regions affected in Alzheimer’s disease is associated with CSF TNF-α in APOE4 carriers. *Neurobiology of Aging*. 2020;86:112-122. doi:10.1016/j.neurobiolaging.2019.10.013

29. D’Anna L, Abu-Rumeileh S, Fabris M, et al. Serum Interleukin-10 Levels Correlate with Cerebrospinal Fluid Amyloid Beta Deposition in Alzheimer Disease Patients. *Neurodegener Dis*. 2017;17(4-5):227-234. doi:10.1159/000474940

30. Davis GK, Baboolal NS, Seales D, Ramchandani J, McKell S, McRae A. Potential biomarkers for dementia in Trinidad and Tobago. *Neuroscience Letters*. 2007;424(1):27-30. doi:10.1016/j.neulet.2007.07.011

31. De Kort AM, Kuiperij HB, Alcolea D, et al. Cerebrospinal fluid levels of the neurotrophic factor neuroleukin are increased in early Alzheimer’s disease, but not in cerebral amyloid angiopathy. *Alz Res Therapy*. 2021;13(1):160. doi:10.1186/s13195-021-00899-0

32. Demirci S, Aynalı A, Demirci K, Demirci S, Arıdoğan BC. The Serum Levels of Resistin and Its Relationship with Other Proinflammatory Cytokines in Patients with Alzheimer’s Disease. *Clin Psychopharmacol Neurosci*. 2017;15(1):59-63. doi:10.9758/cpn.2017.15.1.59

33. Diniz BS, Teixeira AL, Ojopi EB, et al. Higher Serum sTNFR1 Level Predicts Conversion from Mild Cognitive Impairment to Alzheimer’s Disease. *JAD*. 2011;22(4):1305-1311. doi:10.3233/JAD-2010-100921

34. Dukic L, Simundic AM, Martinic-Popovic I, et al. The role of human kallikrein 6, clusterin and adiponectin as potential blood biomarkers of dementia. *Clinical Biochemistry*. 2016;49(3):213-218. doi:10.1016/j.clinbiochem.2015.10.014

35. Dursun E, Gezen-Ak D, Hanağası H, et al. The interleukin 1 alpha, interleukin 1 beta, interleukin 6 and alpha-2-macroglobulin serum levels in patients with early or late onset Alzheimer’s disease, mild cognitive impairment or Parkinson’s disease. *Journal of Neuroimmunology*. 2015;283:50-57. doi:10.1016/j.jneuroim.2015.04.014

36. Edwards M, Hall J, Williams B, Johnson L, O’Bryant S. Molecular Markers of Amnestic Mild Cognitive Impairment among Mexican Americans. Rissman R, ed. *JAD*. 2015;49(1):221-228. doi:10.3233/JAD-150553

37. El-Sayed DA, Salah H, El-Abyary MM, Zaitoun AM, Gaballah A. Alzheimer’s Disease: Serum Biological Markers in Relation to Disease Severity. 2009;46.

38. Forlenza OV, Diniz BS, Talib LL, et al. Increased Serum IL-1β Level in Alzheimer’s Disease and Mild Cognitive Impairment. *Dement Geriatr Cogn Disord*. 2009;28(6):507-512. doi:10.1159/000255051

39. Fu J, Duan J, Mo J, et al. Mild Cognitive Impairment Patients Have Higher Regulatory T-Cell Proportions Compared With Alzheimer’s Disease-Related Dementia Patients. *Front Aging Neurosci*. 2021;12:624304. doi:10.3389/fnagi.2020.624304

40. Fu K, Chiu M, Wara‐aswapati N, et al. Oral microbiome and serological analyses on association of Alzheimer’s disease and periodontitis. *Oral Diseases*. Published online August 30, 2022:odi.14348. doi:10.1111/odi.14348

41. Galgani A, Vergallo A, Campese N, et al. Biological determinants of blood‐based cytokines in the Alzheimer’s disease clinical continuum. *Journal of Neurochemistry*. 2022;163(1):40-52. doi:10.1111/jnc.15686

42. Galimberti D, Venturelli E, Fenoglio C, et al. Intrathecal levels of IL-6, IL-11 and LIF in Alzheimer’s disease and frontotemporal lobar degeneration. *J Neurol*. 2008;255(4):539-544. doi:10.1007/s00415-008-0737-6

43. Gangishetti U, Christina Howell J, Perrin RJ, et al. Non-beta-amyloid/tau cerebrospinal fluid markers inform staging and progression in Alzheimer’s disease. *Alz Res Therapy*. 2018;10(1):98. doi:10.1186/s13195-018-0426-3

44. Gezen-Ak D, Dursun E, Hanağası H, et al. BDNF, TNFα, HSP90, CFH, and IL-10 Serum Levels in Patients with Early or Late Onset Alzheimer’s Disease or Mild Cognitive Impairment. *JAD*. 2013;37(1):185-195. doi:10.3233/JAD-130497

45. Giacconi R, D’Aquila P, Balietti M, et al. Bacterial DNAemia in Alzheimer’s Disease and Mild Cognitive Impairment: Association with Cognitive Decline, Plasma BDNF Levels, and Inflammatory Response. *IJMS*. 2022;24(1):78. doi:10.3390/ijms24010078

46. Gispert JD, Monté GC, Falcon C, et al. CSF YKL-40 and pTau181 are related to different cerebral morphometric patterns in early AD. *Neurobiology of Aging*. 2016;38:47-55. doi:10.1016/j.neurobiolaging.2015.10.022

47. Gongora-Rivera F, Gonzalez-Aquines A, Ortiz-Jiménez X, de la Garza CM, Salinas-Carmona M. Chemokine profile in Alzheimer’s disease: Results from a Mexican population. *Journal of Clinical Neuroscience*. 2020;73:159-161. doi:10.1016/j.jocn.2019.12.051

48. Guo M, Li J, Wang P. Correlation of the expression of NLRP3 mNRA inflammasome and its downstream inflammatory factors in peripheral blood with cognition and activity of daily living of patients with Alzheimer’s disease. :10.

49. Hao J, Qiao Y, Li T, et al. Investigating Changes in the Serum Inflammatory Factors in Alzheimer’s Disease and Their Correlation with Cognitive Function. *JAD*. 2021;84(2):835-842. doi:10.3233/JAD-210552

50. Hesse R, Wahler A, Gummert P, et al. Decreased IL-8 levels in CSF and serum of AD patients and negative correlation of MMSE and IL-1β. *BMC Neurol*. 2016;16(1):185. doi:10.1186/s12883-016-0707-z

51. Italiani P, Puxeddu I, Napoletano S, et al. Circulating levels of IL-1 family cytokines and receptors in Alzheimer’s disease: new markers of disease progression? *J Neuroinflammation*. 2018;15(1):342. doi:10.1186/s12974-018-1376-1

52. Ju T, Sun L, Fan Y, et al. Decreased Netrin-1 in Mild Cognitive Impairment and Alzheimer’s Disease Patients. *Front Aging Neurosci*. 2022;13:762649. doi:10.3389/fnagi.2021.762649

53. Kálmán J, Juhász A, Laird G, et al. Serum interleukin-6 levels correlate with the severity of dementia in Down syndrome and in Alzheimer’s disease. *Acta Neurologica Scandinavica*. 2009;96(4):236-240. doi:10.1111/j.1600-0404.1997.tb00275.x

54. Kamer AR, Craig RG, Pirraglia E, et al. TNF-α and antibodies to periodontal bacteria discriminate between Alzheimer’s disease patients and normal subjects. *Journal of Neuroimmunology*. 2009;216(1-2):92-97. doi:10.1016/j.jneuroim.2009.08.013

55. Kassner S, Bonaterra G, Kaiser E, et al. Novel Systemic Markers for Patients with Alzheimer Disease? – A Pilot Study. *CAR*. 2008;5(4):358-366. doi:10.2174/156720508785132253

56. Khedr EM, Gomaa AMS, Ahmed OG, Sayed HMM, Gamea A. Cognitive Impairment, P300, and Transforming Growth Factor β1 in Different Forms of Dementia. *JAD*. 2020;78(2):837-845. doi:10.3233/JAD-200885

57. Kim SM, Song J, Kim S, et al. Identification of peripheral inflammatory markers between normal control and Alzheimer’s disease. *BMC Neurol*. 2011;11(1):51. doi:10.1186/1471-2377-11-51

58. King E, O’Brien JT, Donaghy P, et al. Peripheral inflammation in mild cognitive impairment with possible and probable Lewy body disease and Alzheimer’s disease. *Int Psychogeriatr*. 2019;31(04):551-560. doi:10.1017/S1041610218001126

59. Koutentaki E, Basta M, Antypa D, et al. IL-6 Enhances the Negative Impact of Cortisol on Cognition among Community-Dwelling Older People without Dementia. *Healthcare*. 2023;11(7):951. doi:10.3390/healthcare11070951

60. Lawlor BA, Swanwick GRJ, Feighery C, Walsh JB, Coakley D. Acute Phase Reactants in Alzheimer’s Disease. Published online 1996.

61. Le Page A, Garneau H, Dupuis G, et al. Differential Phenotypes of Myeloid-Derived Suppressor and T Regulatory Cells and Cytokine Levels in Amnestic Mild Cognitive Impairment Subjects Compared to Mild Alzheimer Diseased Patients. *Front Immunol*. 2017;8:783. doi:10.3389/fimmu.2017.00783

62. Liang CS, Su KP, Tsai CL, et al. The role of interleukin-33 in patients with mild cognitive impairment and Alzheimer’s disease. *Alz Res Therapy*. 2020;12(1):86. doi:10.1186/s13195-020-00652-z

63. Liang CS, Tsai CL, Lin GY, et al. Better Identification of Cognitive Decline With Interleukin-2 Than With Amyloid and Tau Protein Biomarkers in Amnestic Mild Cognitive Impairment. *Front Aging Neurosci*. 2021;13:670115. doi:10.3389/fnagi.2021.670115

64. Llano DA, Li J, Waring JF, et al. Cerebrospinal Fluid Cytokine Dynamics Differ Between Alzheimer Disease Patients and Elderly Controls. *Alzheimer Disease & Associated Disorders*. 2012;26(4):322-328. doi:10.1097/WAD.0b013e31823b2728

65. Lleó A, Alcolea D, Martínez‐Lage P, et al. Longitudinal cerebrospinal fluid biomarker trajectories along the Alzheimer’s disease continuum in the BIOMARKAPD study. *Alzheimer’s &amp; Dementia*. 2019;15(6):742-753. doi:10.1016/j.jalz.2019.01.015

66. Lourenco MV, Ribeiro FC, Santos LE, et al. Cerebrospinal Fluid Neurotransmitters, Cytokines, and Chemokines in Alzheimer’s and Lewy Body Diseases. Maccioni R, ed. *JAD*. 2021;82(3):1067-1074. doi:10.3233/JAD-210147

67. Lu G, Liu W, Huang X, Zhao Y. Complement factor H levels are decreased and correlated with serum C-reactive protein in late-onset Alzheimer’s disease. *Arq Neuro-Psiquiatr*. 2020;78(2):76-80. doi:10.1590/0004-282x20190151

68. Marksteiner J, Kemmler G, Weiss EM, et al. Five out of 16 plasma signaling proteins are enhanced in plasma of patients with mild cognitive impairment and Alzheimer’s disease. *Neurobiology of Aging*. 2011;32(3):539-540. doi:10.1016/j.neurobiolaging.2009.03.011

69. Malaguarnera L, Motta M, Di Rosa M, Anzaldi M, Malaguarnera M. Interleukin-18 and transforming growth factor-beta 1 plasma levels in Alzheimer’s disease and vascular dementia. *Neuropathology*. 2006;26(4):307-312. doi:10.1111/j.1440-1789.2006.00701.x

70. Motta M, Imbesi R, Di Rosa M, Stivala F, Malaguarnera L. Altered plasma cytokine levels in Alzheimer’s disease: Correlation with the disease progression. *Immunology Letters*. 2007;114(1):46-51. doi:10.1016/j.imlet.2007.09.002

71. Muszyński P, Kulczyńska-Przybik A, Borawska R, et al. The Relationship between Markers of Inflammation and Degeneration in the Central Nervous System and the Blood-Brain Barrier Impairment in Alzheimer’s Disease. Lewczuk P, ed. *JAD*. 2017;59(3):903-912. doi:10.3233/JAD-170220

72. Nie J, Fang Y, Chen Y, et al. Characteristics of Dysregulated Proinflammatory Cytokines and Cognitive Dysfunction in Late-Life Depression and Amnestic Mild Cognitive Impairment. *Front Immunol*. 2022;12:803633. doi:10.3389/fimmu.2021.803633

73. Nordengen K, Kirsebom BE, Henjum K, et al. Glial activation and inflammation along the Alzheimer’s disease continuum. *J Neuroinflammation*. 2019;16(1):46. doi:10.1186/s12974-019-1399-2

74. Park JK, Lee KJ, Kim JY, Kim H. The Association of Blood-Based Inflammatory Factors IL-1β, TGF-β and CRP with Cognitive Function in Alzheimer’s Disease and Mild Cognitive Impairment. *Psychiatry Investig*. 2021;18(1):11-18. doi:10.30773/pi.2020.0205

75. Petersen M, Hall J, Parsons T, Johnson L, O’Bryant S. Combining Select Blood-Based Biomarkers with Neuropsychological Assessment to Detect Mild Cognitive Impairment among Mexican Americans. *JAD*. 2020;75(3):739-750. doi:10.3233/JAD-191264

76. Porcellini E, Davis EJ, Chiappelli M, et al. Elevated Plasma Levels of -1-Anti-Chymotrypsin in Age-Related Cognitive Decline and Alzheimer’s Disease: A Potential Therapeutic Target.

77. Reale M, Kamal MA, Velluto L, Gambi D, Nicola MD, Greig NH. Relationship between Inflammatory Mediators, Aβ Levels and ApoE Genotype in Alzheimer Disease.

78. Rizzi L, Roriz-Cruz M. Cerebrospinal fluid inflammatory markers in amnestic mild cognitive impairment: CSF inflammatory markers in aMCI. *Geriatr Gerontol Int*. 2017;17(2):239-245. doi:10.1111/ggi.12704

79. Rota E, Bellone G, Rocca P, Bergamasco B, Emanuelli G, Ferrero P. Increased intrathecal TGF-β1, but not IL-12, IFN-γ and IL-10 levels in Alzheimer’s disease patients. *Neurol Sci*. 2006;27(1):33-39. doi:10.1007/s10072-006-0562-6

80. Rui W, Xiao H, Fan Y, et al. Systemic inflammasome activation and pyroptosis associate with the progression of amnestic mild cognitive impairment and Alzheimer’s disease. *J Neuroinflammation*. 2021;18(1):280. doi:10.1186/s12974-021-02329-2

81. Savaş S. Determination of adiponectin, tumor necrosis factor-alpha, and adhesion molecules in Azheimer’s disease. *Erciyes Med J*. Published online 2019. doi:10.14744/etd.2019.60420

82. Shen XN, Lu Y, Tan CTY, et al. Identification of inflammatory and vascular markers associated with mild cognitive impairment. *Aging*. 2019;11(8):2403-2419. doi:10.18632/aging.101924

83. Shen H, Han C, Yang Y, et al. Pyroptosis executive protein GSDMD as a biomarker for diagnosis and identification of Alzheimer’s disease. *Brain Behav*. 2021;11(4). doi:10.1002/brb3.2063

84. Smith ER, Nilforooshan R, Weaving G, Tabet N. Plasma Fetuin-A is Associated with the Severity of Cognitive Impairment in Mild-to-Moderate Alzheimer’s Disease. *JAD*. 2011;24(2):327-333. doi:10.3233/JAD-2011-101872

85. Song IU, Chung SW, Kim YD, Maeng LS. Relationship between the hs-CRP as non-specific biomarker and Alzheimer’s disease according to aging process. *Int J Med Sci*. 2015;12(8):613-617. doi:10.7150/ijms.12742

86. Startin CM, Ashton NJ, Hamburg S, et al. Plasma biomarkers for amyloid, tau, and cytokines in Down syndrome and sporadic Alzheimer’s disease. *Alz Res Therapy*. 2019;11(1):26. doi:10.1186/s13195-019-0477-0

87. Sun P, Shi S, Wang L, et al. The correlative analysis of IL-1β, IL-6, TNF-α, Hcy,. :5.

88. Sun Q, Ni J, Wei M, et al. Plasma β-amyloid, tau, neurodegeneration biomarkers and inflammatory factors of probable Alzheimer’s disease dementia in Chinese individuals. *Front Aging Neurosci*. 2022;14:963845. doi:10.3389/fnagi.2022.963845

89. Teunissen CE, Lütjohann D, Von Bergmann K, et al. Combination of serum markers related to several mechanisms in Alzheimer’s disease. *Neurobiology of Aging*. 2003;24(7):893-902. doi:10.1016/S0197-4580(03)00005-8

90. Tisato V, Rimondi E, Brombo G, et al. Serum Soluble Tumor Necrosis Factor-Related Apoptosis-Inducing Ligand Levels in Older Subjects with Dementia and Mild Cognitive Impairment. *Dement Geriatr Cogn Disord*. 2016;41(5-6):273-280. doi:10.1159/000446275

91. Villarreal AE, O’Bryant SE, Edwards M, Grajales S, Britton GB, Panama Aging Research Initiative. Serum-based protein profiles of Alzheimer’s disease and mild cognitive impairment in elderly Hispanics. *Neurodegenerative Disease Management*. 2016;6(3):203-213. doi:10.2217/nmt-2015-0009

92. Vurgun U. Association of peripheral visfatin levels and visfatin G-948T gene polymorphism with Alzheimer’s Disease in a Turkish population. *Turkish Journal of Geriatrics*. 2013;16(2):5.

93. Walker KA, Gross AL, Moghekar AR, et al. Association of peripheral inflammatory markers with connectivity in large-scale functional brain networks of non-demented older adults. *Brain, Behavior, and Immunity*. 2020;87:388-396. doi:10.1016/j.bbi.2020.01.006

94. Wang L. Cerebrospinal fluid levels of YKL-40 in prodromal Alzheimer’s disease. *Neuroscience Letters*. Published online 2020:5.

95. Wang T, Xiao S, Liu Y, et al. The efficacy of plasma biomarkers in early diagnosis of Alzheimer’s disease: Biomarkers for Alzheimer’s disease diagnosis. *Int J Geriatr Psychiatry*. 2014;29(7):713-719. doi:10.1002/gps.4053

96. Wennberg AMV, Hagen CE, Machulda MM, Knopman DS, Petersen RC, Mielke MM. The Cross-sectional and Longitudinal Associations Between IL-6, IL-10, and TNFα and Cognitive Outcomes in the Mayo Clinic Study of Aging. *The Journals of Gerontology: Series A*. 2019;74(8):1289-1295. doi:10.1093/gerona/gly217

97. Wennström M, Hall S, Nägga K, Londos E, Minthon L, Hansson O. Cerebrospinal fluid levels of IL-6 are decreased and correlate with cognitive status in DLB patients. *Alz Res Therapy*. 2015;7(1):63. doi:10.1186/s13195-015-0145-y

98. Wennström M, Surova Y, Hall S, et al. The Inflammatory Marker YKL-40 Is Elevated in Cerebrospinal Fluid from Patients with Alzheimer’s but Not Parkinson’s Disease or Dementia with Lewy Bodies. Bartres-Faz D, ed. *PLoS ONE*. 2015;10(8):e0135458. doi:10.1371/journal.pone.0135458

99. Wennström M, Nielsen HM, Orhan F, Londos E, Minthon L, Erhardt S. Kynurenic Acid Levels in Cerebrospinal Fluid from Patients with Alzheimer’s Disease or Dementia with Lewy Bodies. *Int J Tryptophan Res*. 2014;7:IJTR.S13958. doi:10.4137/IJTR.S13958

100. Xin Y, Zhang L, Hu J, Gao H, Zhang B. Correlation of early cognitive dysfunction with inflammatory factors and metabolic indicators in patients with Alzheimer’s disease. *Am J Transl Res*. 2021;13(8):8.

101. Xu Y, Shen Y ‐Y., Zhang X ‐P., et al. Diagnostic potential of urinary monocyte chemoattractant protein‐1 for Alzheimer’s disease and amnestic mild cognitive impairment. *Eur J Neurol*. 2020;27(8):1429-1435. doi:10.1111/ene.14254

102. Yamamoto H, Watanabe T, Miyazaki A, et al. High Prevalence of Chlamydia Pneumoniae Antibodies and Increased High-Sensitive C-Reactive Protein in Patients with Vascular Dementia: CHLAMYDIA PNEUMONIAE AND VASCULAR DEMENTIA. *Journal of the American Geriatrics Society*. 2005;53(4):583-589. doi:10.1111/j.1532-5415.2005.53204.x

103. Yarchoan M, Louneva N, Xie SX, et al. Association of plasma C-reactive protein levels with the diagnosis of Alzheimer’s disease. *Journal of the Neurological Sciences*. 2013;333(1-2):9-12. doi:10.1016/j.jns.2013.05.028

104. Yeram N, Dalvi S, Mankeshwar R, et al. Relationship between cortisol, Interleukin-6 and homocysteine in Alzheimer’s disease. *Qatar Medical Journal*. 2021;2021(2). doi:10.5339/qmj.2021.33

105. Yu S, Liu YP, Liu HL, et al. Serum Protein-Based Profiles as Novel Biomarkers for the Diagnosis of Alzheimer’s Disease. *Mol Neurobiol*. Published online May 31, 2017. doi:10.1007/s12035-017-0609-0

106. Zhao SJ, Guo CN, Wang MQ, Chen WJ, Zhao YB. Serum levels of inflammation factors and cognitive performance in amnestic mild cognitive impairment: A Chinese clinical study. *Cytokine*. 2012;57(2):221-225. doi:10.1016/j.cyto.2011.11.006

107. Zhou X, Wang Z, Xu J, et al. Study on the changes in serum C-reactive protein and amyloid protein levels in elderly patients with suspected cognitive impairment. :8.

108. Zorkina Y, Abramova O, Ushakova V, et al. Inflammatory biomarkers and lipid metabolism parameters in women with mild cognitive impairment and dementia. *Women & Health*. 2023;63(4):285-295. doi:10.1080/03630242.2023.2185750

109. Zuliani G, Ranzini M, Guerra G, et al. Plasma cytokines profile in older subjects with late onset Alzheimer’s disease or vascular dementia. *Journal of Psychiatric Research*. 2007;41(8):686-693. doi:10.1016/j.jpsychires.2006.02.008

110. Zuliani G, Cavalieri M, Galvani M, et al. Markers of endothelial dysfunction in older subjects with late onset Alzheimer’s disease or vascular dementia. *Journal of the Neurological Sciences*. 2008;272(1-2):164-170. doi:10.1016/j.jns.2008.05.020

111. Zuliani G, Marsillach J, Trentini A, Rosta V, Cervellati C. Lipoprotein-Associated Phospholipase A2 Activity as Potential Biomarker of Vascular Dementia. *Antioxidants*. 2023;12(3):597. doi:10.3390/antiox12030597

112. Rider P, Carmi Y, Voronov E, Apte RN. Interleukin-1α. *Seminars in Immunology*. 2013;25(6):430-438. doi:10.1016/j.smim.2013.10.005

113. Basu A, Krady JK, Levison SW. Interleukin‐1: A master regulator of neuroinflammation. 2004;(78):151-156.

114. Nelms K, Keegan AD, Zamorano J, Ryan JJ, Paul WE. THE IL-4 RECEPTOR: Signaling Mechanisms and Biologic Functions. *Annu Rev Immunol*. 1999;17(1):701-738. doi:10.1146/annurev.immunol.17.1.701

115. Tang RH, Qi RQ, Liu HY. Interleukin-4 affects microglial autophagic flux. *Neural Regen Res*. 2019;14(9):1594. doi:10.4103/1673-5374.255975

116. Choy E, Rose-John S. Interleukin-6 as a Multifunctional Regulator: Inflammation, Immune Response, and Fibrosis. *Journal of Scleroderma and Related Disorders*. 2017;2(2_suppl):S1-S5. doi:10.5301/jsrd.5000265

117. West PK, Viengkhou B, Campbell IL, Hofer MJ. Microglia responses to interleukin‐6 and type I interferons in neuroinflammatory disease. *Glia*. 2019;67(10):1821-1841. doi:10.1002/glia.23634

118. Erta M, Quintana A, Hidalgo J. Interleukin-6, a Major Cytokine in the Central Nervous System. *Int J Biol Sci*. 2012;8(9):1254-1266. doi:10.7150/ijbs.4679

119. Matsushima K, Yang D, Oppenheim JJ. Interleukin-8: An evolving chemokine. *Cytokine*. 2022;153:155828. doi:10.1016/j.cyto.2022.155828

120. Tsai SJ. Role of interleukin 8 in depression and other psychiatric disorders. *Progress in Neuro-Psychopharmacology and Biological Psychiatry*. 2021;106:110173. doi:10.1016/j.pnpbp.2020.110173

121. Ihim SA, Abubakar SD, Zian Z, et al. Interleukin-18 cytokine in immunity, inflammation, and autoimmunity: Biological role in induction, regulation, and treatment. *Front Immunol*. 2022;13:919973. doi:10.3389/fimmu.2022.919973

122. Ojala J, Sutinen E. The Role of Interleukin-18, Oxidative Stress and Metabolic Syndrome in Alzheimer’s Disease. *JCM*. 2017;6(5):55. doi:10.3390/jcm6050055

123. Sproston NR, Ashworth JJ. Role of C-Reactive Protein at Sites of Inflammation and Infection. *Front Immunol*. 2018;9:754. doi:10.3389/fimmu.2018.00754

124. Strang F, Scheichl A, Chen YC, et al. Amyloid Plaques Dissociate Pentameric to Monomeric C-Reactive Protein: A Novel Pathomechanism Driving Cortical Inflammation in Alzheimer’s Disease?: mCRP in Alzheimer’s Disease. *Brain Pathology*. 2012;22(3):337-346. doi:10.1111/j.1750-3639.2011.00539.x

125. Croft M. The role of TNF superfamily members in T-cell function and diseases. *Nat Rev Immunol*. 2009;9(4):271-285. doi:10.1038/nri2526

126. Muhammad M. Tumor necrosis factor alpha: a major cytokine of brain neuroinflammation. *Cytokines*. 2019;861231.

127. Sharma V, Thakur V, Singh SN, Guleria R. Tumor Necrosis Factor and Alzheimer’s Disease: A Cause and Consequence Relationship. *Klinik Psikofarmakoloji Bülteni-Bulletin of Clinical Psychopharmacology*. 2012;22(1):86-97. doi:10.5455/bcp.20120112064639

128. Deshmane SL, Kremlev S, Amini S, Sawaya BE. Monocyte Chemoattractant Protein-1 (MCP-1): An Overview. *Journal of Interferon & Cytokine Research*. 2009;29(6):313-326. doi:10.1089/jir.2008.0027

129. Hinojosa AE, Garcia-Bueno B, Leza JC, Madrigal JL. CCL2/MCP-1 modulation of microglial activation and proliferation. *J Neuroinflammation*. 2011;8(1):77. doi:10.1186/1742-2094-8-77

130. Tizaoui K, Yang JW, Lee KH, et al. The role of YKL-40 in the pathogenesis of autoimmune diseases: a comprehensive review. *Int J Biol Sci*. 2022;18(9):3731-3746. doi:10.7150/ijbs.67587

131. Mavroudis I, Chowdhury R, Petridis F, et al. YKL-40 as a Potential Biomarker for the Differential Diagnosis of Alzheimer’s Disease. *Medicina*. 2021;58(1):60. doi:10.3390/medicina58010060

132. Connolly K, Lehoux M, O’Rourke R, et al. Potential role of chitinase‐3‐like protein 1 (CHI3L1/YKL‐40) in neurodegeneration and Alzheimer’s disease. *Alzheimer’s &amp; Dementia*. 2023;19(1):9-24. doi:10.1002/alz.12612
