# Supplementary material for: “Let's talk about sex, inflammaging, and cognition, baby”: A meta-analysis and meta-regression of 106 case-control studies on mild cognitive impairment and Alzheimer's disease
Source: Brain Behav Immun Health. 2024 Jul 20;40:100819. doi: 10.1016/j.bbih.2024.100819 (PMC11331696; doi:10.1016/j.bbih.2024.100819)
Supplement: Supplementary file 5 [file mmc3.zip › bbih_00100819_Supplementary Material 3_spl_1_spl_.odt]

CRP AD Serum and Plasma

A close up of a ticket

Description automatically generated

Figure S1. Forest plot and funnel plot of serum and plasma C-reactive protein meta-analysis (CRP) in Alzheimer’s disease (AD) and healthy control studies. 

CRP MCI Serum and PlasmaA close-up of a diagram

Description automatically generated

Figure S2. Forest plot and funnel plot of serum and plasma C-reactive protein meta-analysis (CRP) in mild cognitive impairment (MCI) and healthy control studies. 

hsCRP AD Serum and PlasmaA close-up of a document

Description automatically generated

Figure S3. Forest plot and funnel plot of serum and plasma high sensitivity C-reactive protein meta-analysis (hsCRP) in Alzheimer’s disease (AD) and healthy control studies. 

hsCRP MCI Serum and PlasmaA white rectangular object with numbers and lines

Description automatically generated with medium confidence

Figure S4. Forest plot and funnel plot of serum and plasma high sensitivity C-reactive protein (hsCRP) meta-analysis in mild cognitive impairment (MCI) and healthy control studies. 

IFNγ AD Serum and PlasmaA close-up of a computer screen

Description automatically generated

Figure S5. Forest plot and funnel plot of serum and plasma interferon-γ (IFNγ) meta-analysis in Alzheimer’s disease (AD) and healthy control studies. 

IFNγ MCI Serum and PlasmaA close-up of a document

Description automatically generated

Figure S6. Forest plot and funnel plot of serum and plasma interferon-γ (IFNγ) meta-analysis in mild cognitive impairment (MCI) and healthy control studies. 

IL1α AD Serum and PlasmaA close-up of a computer screen

Description automatically generated

Figure S7. Forest plot and funnel plot of serum and plasma interleukin 1α (IL1α) meta-analysis in Alzheimer’s disease (AD) and healthy control studies. 

IL1α MCI Serum and PlasmaA close-up of a check

Description automatically generated

Figure S8. Forest plot and funnel plot of serum and plasma interleukin 1α (ILα) meta-analysis in mild cognitive impairment (MCI) and healthy control studies. 

IL1β AD Serum and PlasmaA graph of a graph

Description automatically generated

Figure S9. Forest plot and funnel plot of serum and plasma interleukin 1β meta-analysis in Alzheimer’s disease (AD) and healthy control studies. 

IL1β MCI Serum and PlasmaA graph of a function

Description automatically generated

Figure S10. Forest plot and funnel plot of serum and plasma interleukin 1β (IL1β) meta-analysis in mild cognitive impairment (MCI) and healthy control studies. 

IL2 AD Serum and PlasmaA close-up of a document

Description automatically generated

Figure S11. Forest plot and funnel plot of serum and plasma interleukin 2 (IL2) meta-analysis in Alzheimer’s disease (AD) and healthy control studies. 

IL2 MCI Serum and PlasmaA close up of a check

Description automatically generated

Figure S12. Forest plot and funnel plot of serum and plasma interleukin 2 (IL2) meta-analysis in mild cognitive impairment (MCI) and healthy control studies. 

IL4 AD Serum and PlasmaA diagram of a graph

Description automatically generated

Figure S13. Forest plot and funnel plot of serum and plasma interleukin 4 (IL4) meta-analysis in Alzheimer’s disease (AD) and healthy control studies. 

IL4 MCI Serum and PlasmaA close-up of a document

Description automatically generated

Figure S14. Forest plot and funnel plot of serum and plasma interleukin 4 (IL4) meta-analysis in mild cognitive impairment (MCI) and healthy control studies. 

IL5 AD Serum and PlasmaA diagram of a graph

Description automatically generated

Figure S15. Forest plot and funnel plot of serum and plasma interleukin 5 (IL5) meta-analysis in Alzheimer’s disease (AD) and healthy control studies. 

IL5 MCI Serum and PlasmaA close-up of a diagram

Description automatically generated

Figure S16. Forest plot and funnel plot of serum and plasma interleukin 5 (IL5) meta-analysis in mild cognitive impairment (MCI) and healthy control studies. 

IL6 AD Serum and Plasma

Figure S17. Forest plot and funnel plot of serum and plasma interleukin 6 (IL6) meta-analysis in Alzheimer’s disease (AD) and healthy control studies. 

IL6 MCI Serum and PlasmaA close-up of a diagram

Description automatically generated

Figure S18. Forest plot and funnel plot of serum and plasma interleukin 6 (IL6) meta-analysis in mild cognitive impairment (MCI) and healthy control studies. 

IL6 AD CSFA white rectangular object with text

Description automatically generated with medium confidence

Figure S19. Forest plot and funnel plot of cerebrospinal fluid (CSF) interleukin 6 (IL6) meta-analysis in Alzheimer’s disease (AD) and healthy control studies. 

IL7 AD Serum and PlasmaA close-up of a document

Description automatically generated

Figure S20. Forest plot and funnel plot of serum and plasma interleukin 7 (IL7) meta-analysis in Alzheimer’s disease (AD) and healthy control studies. 

IL7 MCI Serum and PlasmaA close-up of a computer screen

Description automatically generated

Figure S21. Forest plot and funnel plot of serum and plasma interleukin 7 (IL7) meta-analysis in mild cognitive impairment (MCI) and healthy control studies. 

IL8 AD Serum and PlasmaA close-up of a document

Description automatically generated

Figure S22. Forest plot and funnel plot of serum and plasma interleukin 8 (IL8) meta-analysis in Alzheimer’s disease (AD) and healthy control studies. 

IL8 MCI Serum and Plasma A close-up of a diagram

Description automatically generated

Figure S23. Forest plot and funnel plot of serum and plasma interleukin 8 (IL8) meta-analysis in mild cognitive impairment (MCI) and healthy control studies. 

IL8 AD CSFA close-up of a computer screen

Description automatically generated

Figure S24. Forest plot and funnel plot of cerebrospinal fluid (CSF) interleukin 8 (IL8) meta-analysis in Alzheimer’s disease (AD) and healthy control studies. 

IL10 AD Serum and Plasma A close-up of a graph

Description automatically generated

Figure S25. Forest plot and funnel plot of serum and plasma interleukin 10 (IL10) meta-analysis in Alzheimer’s disease (AD) and healthy control studies. 

IL10 MCI Serum and PlasmaA close up of a document

Description automatically generated

Figure S26. Forest plot and funnel plot of serum and plasma interleukin 10 (IL10) meta-analysis in mild cognitive impairment (MCI) and healthy control studies. 

IL17 AD Serum and PlasmaA close-up of a computer screen

Description automatically generated

Figure S27. Forest plot and funnel plot of serum and plasma interleukin 17 (IL17) meta-analysis in Alzheimer’s disease (AD) and healthy control studies. 

IL18 AD Serum and PlasmaA close up of a check

Description automatically generated

Figure S28. Forest plot and funnel plot of serum and plasma interleukin 18 (IL18) meta-analysis in Alzheimer’s disease (AD) and healthy control studies. 

IL18 MCI Serum and PlasmaA close-up of a diagram

Description automatically generated

Figure S29. Forest plot and funnel plot of serum and plasma interleukin 18 (IL18) meta-analysis in mild cognitive impairment (MCI) and healthy control studies. 

MCP-1 AD Serum and PlasmaA close-up of a check

Description automatically generated

Figure S30. Forest plot and funnel plot of serum and plasma monocyte chemoattractant protein 1 (MCP-1) meta-analysis in Alzheimer’s disease (AD) and healthy control studies. 

MCP-1 MCI Serum and Plasma

Figure S31. Forest plot and funnel plot of serum and plasma monocyte chemoattractant protein 1 (MCP-1) meta-analysis in mild cognitive impairment (MCI) and healthy control studies. 

MCP-1 AD CSFA close-up of a computer screen

Description automatically generated

Figure S32. Forest plot and funnel plot of cerebrospinal fluid (CSF) monocyte chemoattractant protein 1(MCP-1) meta-analysis in Alzheimer’s disease (AD) and healthy control studies. 

MCP-1 MCI CSFA close-up of a computer screen

Description automatically generated

A graph of a graph

Description automatically generated

Figure S33. Forest plot and funnel plot of cerebrospinal fluid (CSF) monocyte chemoattractant protein 1 (MCP-1) meta-analysis in mild cognitive impairment (MCI) and healthy control studies. 

TFGβ AD Serum and PlasmaA close-up of a document

Description automatically generated

Figure S34. Forest plot and funnel plot of serum and plasma tissue growth factor β (TGFβ) meta-analysis in Alzheimer’s disease (AD) and healthy control studies. 

TNFα AD Serum and PlasmaA diagram of numbers and lines

Description automatically generated with medium confidence

Figure S35. Forest plot and funnel plot of serum and plasma tumour necrosis factor α (TNFα) meta-analysis in Alzheimer’s disease (AD) and healthy control studies. 

TNFα MCI Serum and PlasmaA diagram with numbers and lines

Description automatically generated

Figure S36. Forest plot and funnel plot of serum and plasma tumour necrosis factor α (TNFα) meta-analysis in mild cognitive impairment (MCI) and healthy control studies. 

YKL-40 AD CSFA close-up of a white card

Description automatically generated

Figure S37. Forest plot and funnel plot of cerebrospinal fluid (CSF) chitinase-3-like protein 1 (YKL-40) meta-analysis in Alzheimer’s disease (AD) and healthy control studies. 

YKL-40 MCI CSF

Figure S38. Forest plot and funnel plot of cerebrospinal fluid (CSF) chitinase-3-like protein 1 (YKL-40) meta-analysis in mild cognitive impairment (MCI) and healthy control studies. 

Figure S39. The left column depicts the regression outcomes of MMSE from AD and HC studies on inflammatory marker Hedges’ g from serum and plasma (A) IL6, CSF (B) IL6, and serum and plasma (C) IL1α. The right column depicts regression outcomes of MMSE from MCI and HC studies on inflammatory marker Hedges’ g from serum and plasma (D) TNFα and (E) IL8.

Figure S40. The left column depicts the regression outcomes of the proportion of participants who were female in AD and HC studies on inflammatory marker Hedges’ g from serum and plasma (A) IL1α, (B) IL18, and (C) MCP-1. The right column depicts the regression outcomes of the proportion of participants who were female in AD and HC studies on the inflammatory marker Hedges’ g from serum and plasma (D) IL6, (E) hsCRP, (F) IL4, and CSF (G) YKL-40.

Figure S41. The left column depicts the regression outcomes of age from AD and HC on inflammatory marker Hedges’ g from serum and plasma (A) CRP and CSF (B) MCP-1. The right column depicts regression outcomes of age from MCI and HC studies on inflammatory marker Hedge’ g from serum and plasma (D) IL4.
